# Supplementary material for: Structural variant-based pangenome construction has low sensitivity to variability of haplotype-resolved bovine assemblies
Source: Nat Commun. 2022 May 31;13:3012. doi: 10.1038/s41467-022-30680-2 (PMC9156671; doi:10.1038/s41467-022-30680-2)
Supplement: Supplementary file 1 — Supplementary Information [file 41467_2022_30680_MOESM1_ESM.pdf]

Structural variant-based pangenome construction has low sensitivity to variability of haplotype-resolved bovine assemblies

Supplementary Table 1. Sequencing read details for HiFi, ONT, and short reads (SR). Breeding refers to the type of F1 crossing. Target library sizes and SMRT cells are reported for HiFi reads, with multiple libraries separated by commas, as well as the total number of bases, reads, and read N50. ONT data did not have a target library size, but the number of PromethION cells and nuclease washes are reported along with reads, bases, and read N50. Short reads for both the F1s and sires/dams libraries are 150 bp in length, with the number of bases reported.

F1	Breeding	F1 Sex	HiFi library size in kb (cells)	HiFi bases/reads	HiFi N50	ONT cells (nuclease washes)	ONT bases/reads	ONT N50	F1 SR bases	Sire/dam bases
OxO	Within-breed	Female	18.7 (4)	86877013947/ 4491846	19621	2 (0)	97741535249/ 2617673	63993	83420372505	83974454313/ 90279569069
NxB	Inter-subspecies	Male	21 (5), 15 (1)	140232393384/ 6803270	20792	3 (0)	277704422221/ 9066711	44830	61158863267	145456304453/ 152857605524
GxP	Inter-species	Female	10 (9), 15 (4)	139087513200/ 9359887	12914	16 (1)	410851293648/ 15752473	48583	99681429463	97052058593/1 22689575398

Supplementary Table 2. Trio binning of HiFi and ONT long read data. For each F1, the number of heterozygous sites is estimated by aligning short reads to ARS-UCD1.2, calling variants, and counting the number of heterozygous sites. Domintette, the cow on which ARS-UCD1.2 is based, had 2,607,461 heterozygous sites. The percentage of bases/reads assigned to each haplotype, or an unknown bin, are given for each trio. Sep% refers to the separability of reads, i.e. the percentage of F1 bases assigned to either haplotype 1 or 2.

		HiFi				ONT			
F1	Heterozygous sites	Hap1	Hap2	Unknown	Sep%	Hap1	Hap2	Unknown	Sep%
OxO	4,024,944	41.5/40.7	39.6/38.8	18.9/20.5	81.12	51.4/49.8	48.2/47.0	0.4/3.2	99.67
NxB	11,506,532	48.1/48.2	50.7/50.8	1.0/1.2	98.99	46.1/46.7	53.9/52.9	0.0/0.4	99.97
GxP	23,190,285	49.0/49.0	50.9/50.9	0.1/0.1	99.88	47.0/43.4	52.9/48.9	0.1/7.7	99.83

*Supplementary Table 3. Assembly metrics for the additional four assemblers. Columns as are described in Table 1. Due to lower HiFi and ONT coverage for the Original Braunvieh, primary assemblies (i.e., not haplotype-resolved) were assembled where necessary and are marked by *. Assemblies are available online* [1].

Breed or species	Read	Assembler	Size (autosomal size)	Contigs (autosomal contigs)	NG50	PG50	QV	BUSCO (single copy)	Repeat
Original Braunvieh	HiFi	Peregrine*	3.34 (2.56)	3108 (134)	43.6	1.02	48.0	95.7 (93.6)	51.57
		HiCanu*	3.22 (2.56)	1729 (116)	56.4	1.51	51.1	96.2 (94.2)	50.63
		HiCanu	3.03 (2.54)	3240 (822)	7.36	7.22	48.7	95.3 (93.4)	48.01
	ONT	Raven*	2.70 (2.52)	459 (283)	20.0	0.32	37.1	87.3 (85.8)	42.75
		Flye*	2.66 (2.50)	1344 (267)	60.8	0.36	42.8	95.9 (94.2)	42.04
Nellore	HiFi	HiCanu	2.85 (2.52)	3266 (153)	39.2	32.8	44.5	93.4 (91.7)	47.37
		Peregrine	2.91 (2.56)	1302 (127)	38.7	38.3	45.9	92.9 (91.4)	47.31
	ONT	Raven	2.52 (2.50)	185 (131)	62.4	22.3	33.3	86.5 (85.2)	41.43
		Flye	2.53 (2.50)	585 (130)	71.5	25.7	43.0	93.1 (91.6)	41.71
Brown Swiss	HiFi	HiCanu	3.10 (2.51)	3969 (182)	29.0	27.3	43.6	96.1 (94.2)	49.67
		peregrine	3.09 (2.54)	1805 (206)	25.6	22.2	44.9	95.8 (94.1)	49.11
	ONT	Raven	2.74 (2.50)	366 (132)	54.3	36.7	32.3	95.5 (93.9)	43.71
		Flye	2.65 (2.49)	553 (111)	68.2	12.7	42.5	95.7 (94.1)	42.12
gaur	HiFi	HiCanu	2.91 (2.53)	1099 (119)	41.1	39.1	50.3	95.7 (93.9)	46.21
		Peregrine	2.94 (2.52)	2402 (679)	5.7	5.4	46.5	94.8 (93.0)	46.73
	ONT	Raven	2.65 (2.49)	212 (11)	65.8	62.2	36.8	94.8 (93.0)	41.96
		Flye	2.62 (2.48)	345 (145)	68.4	68.2	42.1	93.4 (91.6)	41.86
Piemontese	HiFi	HiCanu	3.03 (2.53)	1575 (119)	47.9	41.9	48.9	95.9 (94.2)	47.81
		Peregrine	3.04 (2.52)	2886 (771)	5.2	4.7	46.5	94.7 (93.0)	47.71
	ONT	Raven	2.74 (2.50)	414 (140)	61.6	61.3	31.9	94.9 (93.2)	43.57
		Flye	2.64 (2.49)	325 (98)	70.0	69.9	44.1	95.7 (94.1)	41.95
Hereford (ARS-UCD1.2)	CLR	Canu	2.72 (2.49)	2597 (289)	25.9	N/A	35.8	95.7 (93.9)	42.96
				VGP Standards	1	0.1	40	90	N/A

Supplementary Table 4. Repetitive content of sequence that was not assigned to chromosomes. Contigs which were scaffolded to unplaced sequence in ARS-UCD1.2 (typically denoted NKL…) are labelled "REF", while contigs which did not align confidently to any existing reference sequence are labelled "ASM". The total number of bases in each contig type are shown in megabases, along with the percentage of repetitive bases identified by RepeatMasker for each major classes, as well as the total percentage of masked bases. The average masked percentage is higher for the 10 produced assemblies compared to ARS-UCD1.2, as expected given the higher potential of HiFi and ONT reads to assemble repetitive regions.

Breed/Species	Assembler	Tigs	Bases (Mb)	SINEs	LINEs	LTR	Satellites	Unclassified	Total masked
Hereford (ARS UCD1.2)	Canu	REF	87.438	2.5	7.9	27.1	51.4	6.7	73.7
gaur	hifiasm	REF	324.624	0.6	0.3	16.2	79.4	3.1	89.9
		ASM	22.850	1.1	1.9	18.5	77.3	3.7	90.0
	Shasta	REF	8.168	0.4	0.9	20.1	79.3	2.3	91.4
		ASM	8.336	3.6	3.5	47.3	32.2	11.1	70.0
Piemontese	hifiasm	REF	295.895	1.1	0.7	22.4	71.4	5.1	86.5
		ASM	97.389	1.8	5.3	28.4	58.6	4.9	78.9
	Shasta	REF	16.705	0.9	3.5	25.6	73.9	4.2	91.1
		ASM	17.535	1.8	5.1	22.7	68.7	5.3	86.2
Nellore	hifiasm	REF	232.134	1.0	0.5	24.4	74.1	5.1	90.0
		ASM	50.400	0.9	2.1	25.8	71.7	3.5	88.2
	Shasta	REF	18.208	0.4	0.9	29.6	74.2	2.1	90.7
		ASM	31.937	1.1	3.8	19.5	74.0	3.8	88.1
Brown Swiss	hifiasm	REF	238.508	1.3	2.7	22.2	66.4	5.5	82.8
		ASM	70.120	0.5	1.0	21.5	80.1	2.0	92.8
	Shasta	REF	43.099	0.7	4.2	22.1	71.3	3.8	86.9
		ASM	22.063	1.5	4.9	21.1	70.3	4.2	86.2
Original Braunvieh	hifiasm	REF	307.811	1.0	0.5	25.6	74.0	4.5	90.2
		ASM	79.682	1.4	3.7	28.3	67.0	4.4	86.1
	Shasta	REF	33.545	0.6	1.6	34.1	70.0	3.6	90.4
		ASM	43.033	1.3	4.4	25.7	69.9	5.0	88.2

Supplementary Table 5. Statistics for hifiasm and Shasta pangenomes. N_b is the total number of bubbles identified in the pangenome. L_{ref} and L_b refer to the mean length of the reference path and non-reference paths through bubbles. N_{Ins} and N_{Del} are respectively the number of predicted insertion or deletion events, for when the reference path is of length zero and non-reference path has non-zero length, or the reverse. K is the mean number of non-reference nodes in bubbles.

	N_b	L_{ref}	L_b	N_{Ins}	N_{Del}	K
hifiasm	98,054	594	1080	27,642	24,456	1.755
Shasta	98,545	584	924	27,512	24,294	1.762

Supplementary Table 6. Pangenome bubbles unique to HiFi or ONT assemblies. Values are averaged across 10 pangenomes constructed from five hifiasm and five Shasta assemblies. Bubbles are considered to be unique to HiFi or ONT if they are 1+ Kb in size and not within 1 Kb of a bubble from the other read technology. Bases are listed in Mb, with the repeat types given as percentages.

Read	Bubbles	Bases (Mb)	SINEs	LINEs	LTR	Satellites	Unclassified	Total Masked
HiFi	57	1.85	5.9	22.9	5.8	4.3	13.0	54.0
ONT	36	0.39	8.4	12.9	4.0	3.8	14.9	45.3

*Supplementary Table 7. Quality metrics for downsampled assemblies. Coverage for hifiasm assemblies is diploid (denoted by *), while Shasta coverage is the trio binned coverage for the respective haplotype (except for Original Braunvieh, which is diploid coverage followed by polishing-based phasing).*

Breed/species	Read technology	Coverage	Size (autosomal size)	Contigs (autosomal contigs)	NG50	PG50	QV	BUSCO (single copy)	Repeat
Original Braunvieh	hifiasm	19.3*	2.96 (2.53)	3266 (1654)	2.9	2.3	43.5	92.7 (90.3)	46.5
	Shasta	29.0	2.70 (2.48)	2104 (114)	50.6	2.3	39.8	95.1 (93.4)	43.2
Brown Swiss	hifiasm	20.3*	2.92 (2.53)	3015 (1789)	2.3	2.2	40.7	93.7 (92.1)	46.4
	Shasta	24.4	2.68 (2.48)	2456 (418)	10.4	10.0	40.0	94.7 (93.1)	43.0
Nellore	hifiasm	20.3*	2.81 (2.54)	3016 (1863)	2.2	2.2	40.9	91.1 (89.6)	45.9
	Shasta	20.9	2.55 (2.48)	2399 (399)	10.9	10.8	40.1	92.5 (91.0)	42.8
gaur	hifiasm	24.2*	2.90 (2.50)	4768 (2055)	2.0	1.8	42.4	94.1 (92.3)	45.8
	Shasta	23.6	2.64 (2.48)	1065 (411)	13.5	13.5	38.4	94.1 (92.3)	42.0
Pied	hifiasm	24.2*	2.97 (2.51)	5176 (2128)	1.9	1.7	42.3	94.0 (92.3)	46.5
	Shasta	26.5	2.63 (2.47)	1459 (419)	11.6	11.6	37.9	94.3 (92.3)	42.2

Supplementary Table 8. NG50 for merged hifiasm and Shasta assemblies. Assemblies were merged with RagTag for each assembly pair separately, either merging the Shasta assembly into the hifiasm assembly (Merge [HiFi-backed]) or vice versa. Contig NG50 values are given in Mb.

	Breeds/species					
	O (pat)	O (mat)	N	B	G	P
HiFi	56.0	47.0	94.4	86.7	73.5	52.0
ONT	71.6	71.7	68.5	64.0	68.1	82.8
Merge (HiFi-backed)	62.4	65.6	96.4	86.7	73.5	65.2
Merge (ONT-backed)	72.9	72.9	68.5	64.0	73.7	84.1

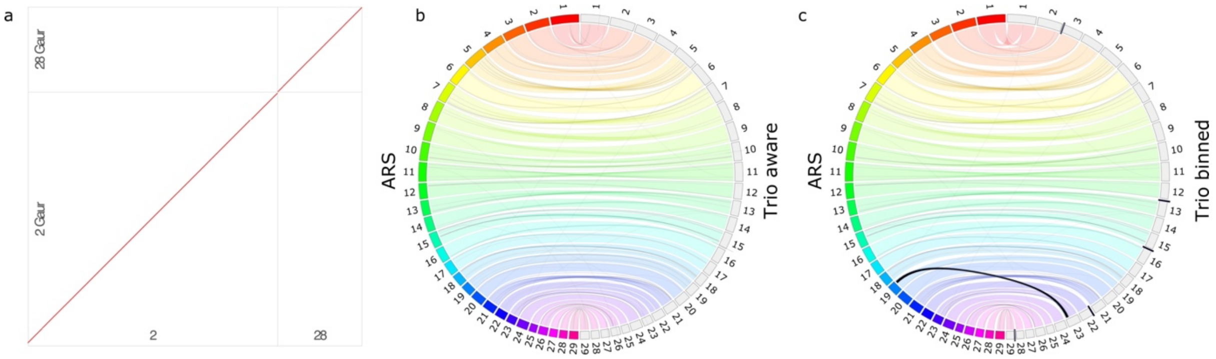

Supplementary Figure 1. Alignment plots of hifiasm gaur assemblies and ARS-UCD1.2. a) Dot plot of chromosomes 2 and 28 in gaur (left) and ARS-UCD1.2 (bottom). Since the gaur assembly did not span the centromeric fusion, the scaffolding to chromosomes 2 and 28 behave normally. b) The trio aware assembly of the GxP gaur haplotype has no major misassemblies compared to ARS-UCD1.2, while the trio binned assembly in c) has several, like 20 Mb of sequence from chromosome 19 is assembled into chromosome 23. Similarly, the trio binned assembly has greater standard deviation in autosome size compared to ARS-UCD1.2 than the trio aware assembly (±6.2 versus 1.4 Mb). After accounting for differences in centromeric sequence, the trio binned gaur assembly had more than 5 Mb of sequence potentially missing on chromosomes 3 and 29, while it also had more than 5 Mb of potentially spurious sequence on chromosomes 13, 16, and 22. The trio binned assembly does have more autosomal centromeric sequence (44 versus 17 Mb) but appears to be missing nearly 23 Mb of non-centromeric sequence (9 Mb of which is repetitive). This is reflected in the higher BUSCO score for the trio aware mode compared to trio binned (95.7% versus 94.2% complete BUSCOs).

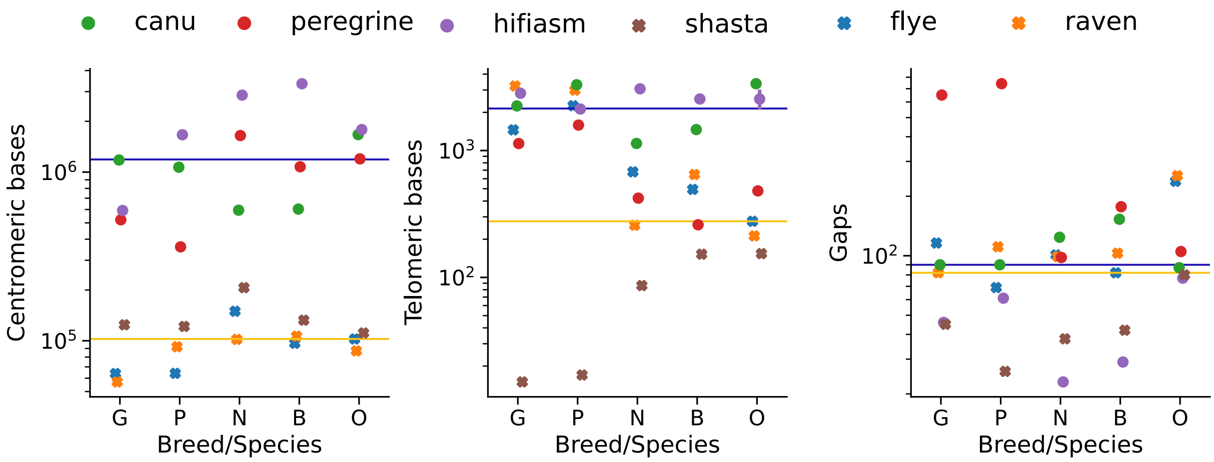

Supplementary Figure 2. Centromere and telomere completeness of assemblies. HiFi-based assemblies (circles) generally outperform ONT-based assemblies (crosses) for mean centromere and telomere completeness across the n=29 autosomes for the different breeed/species assembled. However, ONT-based assemblies have slightly fewer gaps. Error bars represent the 95% confidence interval Mean values across each sequencing technology are marked by blue (HiFi) and orange (ONT) lines. Shasta tends to under-perform relative to the ONT average for telomere completeness, potentially due to the marker-graph assembly strategy, while hifiasm tends to over-perform relative to HiFi average for the number of autosome gaps.

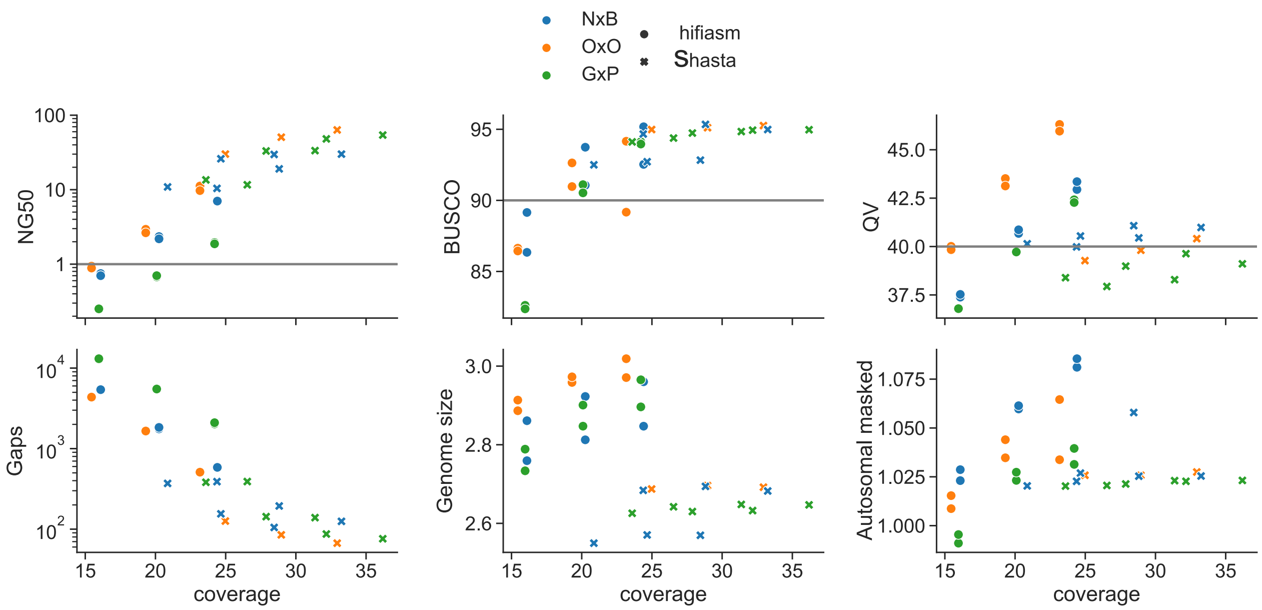

Supplementary Figure 3. Assembly quality metrics for "medium" coverage. The metrics are the same as described in Figure 3. Results are overall similar between the three F1s, where approximately 20x diploid HiFi coverage or 30x haploid ONT coverage is sufficient to produce assemblies at or above the VGP quality standards.

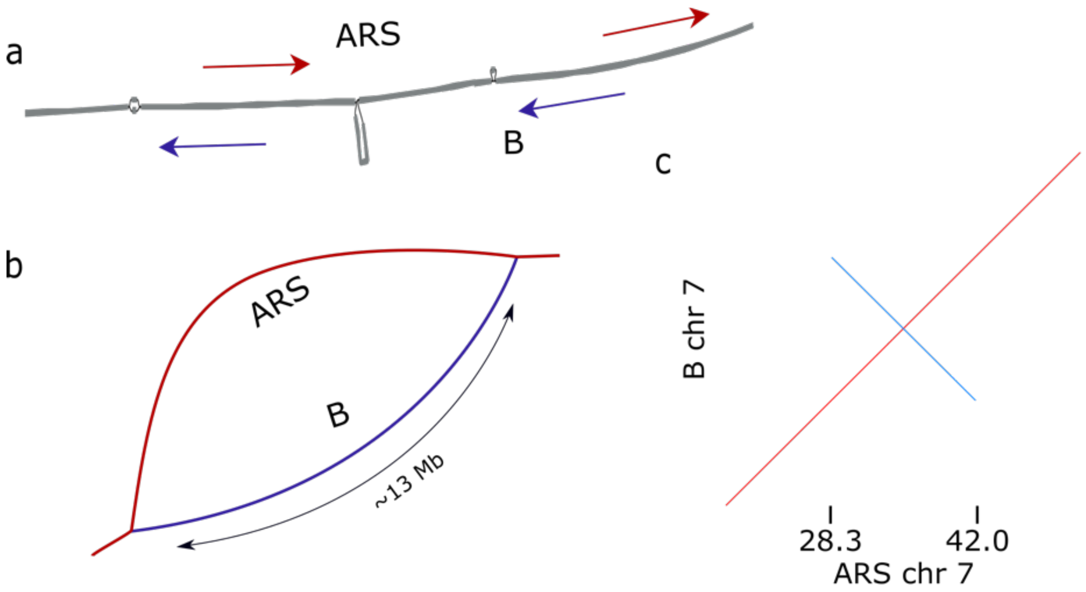

Supplementary Figure 4. Palindromic inversion on BTA7 in pangenome. Minigraph either maps chromosome 7 of the hifiasm assembly of the Brown Swiss haplotype as a) the negative strand (blue arrows) of many small existing nodes in the graph (red arrows) or b) one large, inverted bubble. The latter case generally happens when the Brown Swiss hifiasm assembly is added first. c) A dot-plot of this region aligned between ARS-UCD1.2 and the Brown Swiss hifiasm assembly indicate the region between BTA7:28.3-42.0 Mb is palindromic with support for forward (red) and reverse (blue) alignments. The alignment score is only slightly higher for the reverse alignment compared to the forward, corresponding to graph cases a) and b) respectively.

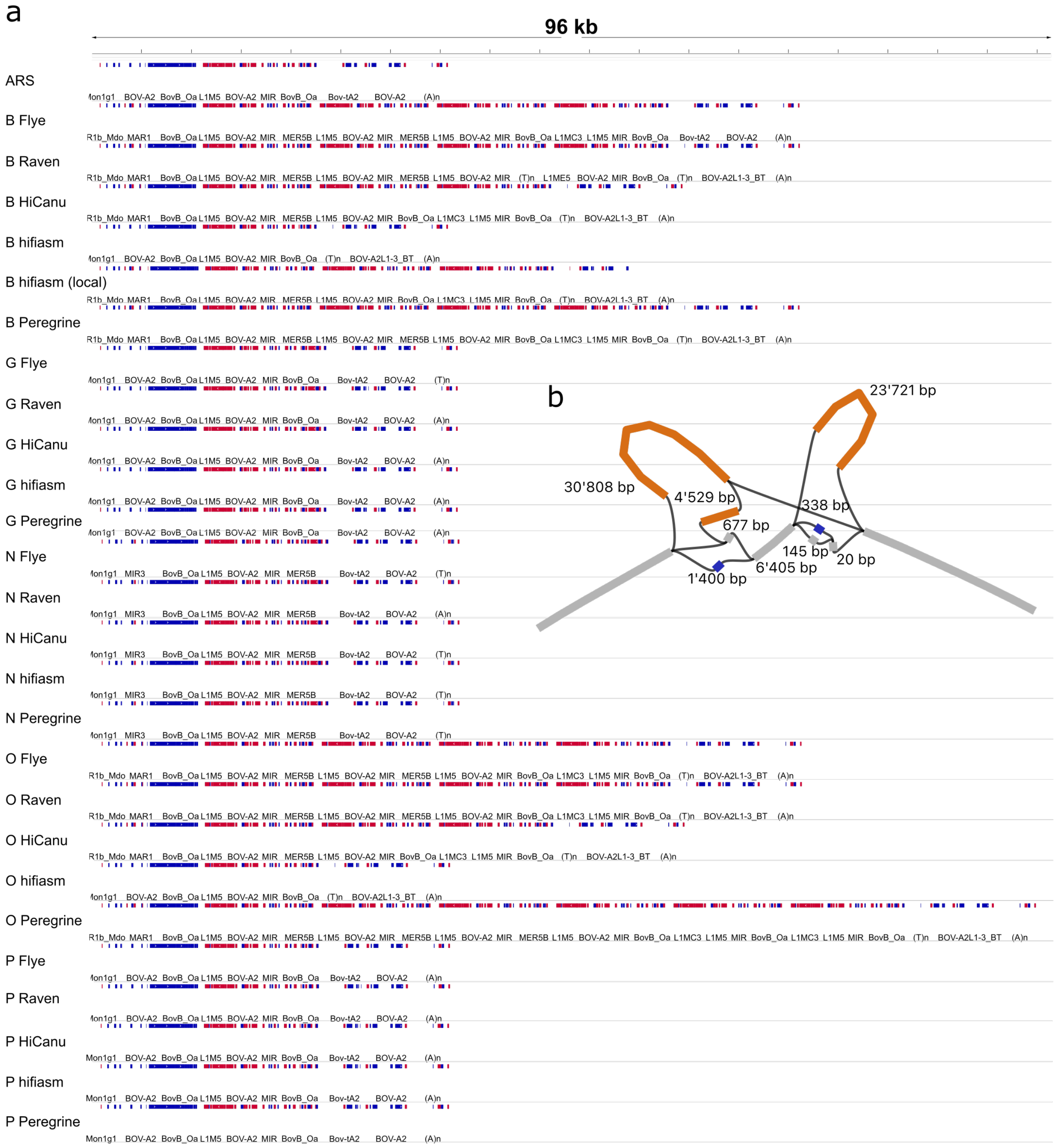

Supplementary Figure 5. GC SV copy number is highly inconsistent across assemblers. a) The same repeat analysis as described in Figure 6 confirms the tandem duplication is never encountered in gaur (G), Nellore (N), and Piemontese (P) across all examined assemblers. However, the duplication copy number for Original Braunvieh ranges from zero to five, while Brown Swiss (B) ranges from zero to three copies. A local re-assembly of the relevant Brown Swiss HiFi reads (align reads to ARS-UCD1.2, and then extract all reads in a ±20 Kb interval around the ARS-UCD1.2 coordinates of the SV) with hifiasm produces two copies, confirming the ability to resolve this CNV is not a limitation of hifiasm but potentially a sensitivity issue when assembling the whole genome. b) The 31 assembly pangenome still identifies the CNV as well as the 200 bp and 700 bp insertions in gaur and Nellore.

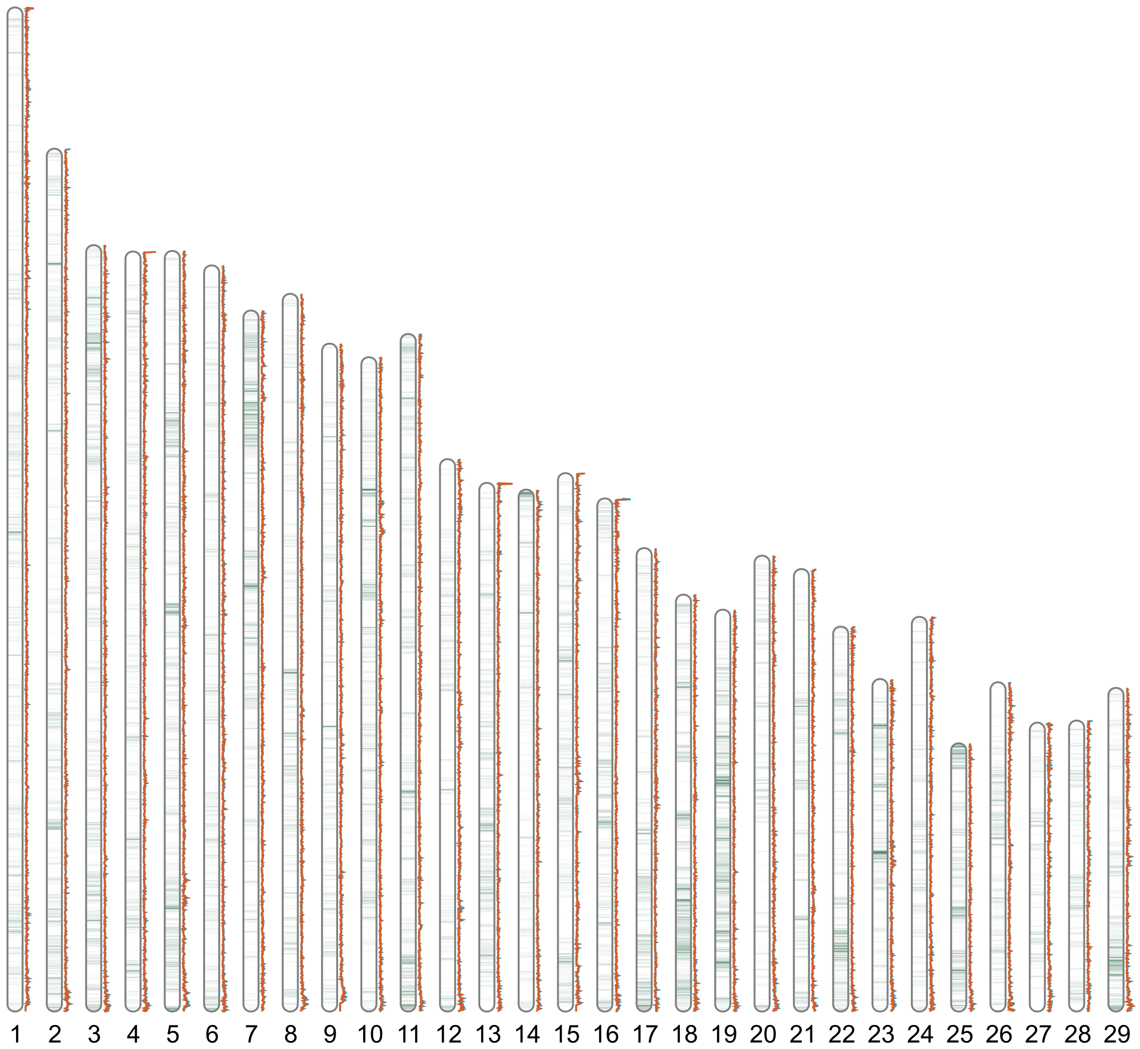

Supplementary Figure 6. Overlaps between protein coding regions and pangenome bubbles. The number of CDS regions in the ARS-UCD1.2 RefSeq 106 annotation per 100 Kb window are shown as a heatmap (white – low to green – high) along the autosomes. Similarly, the number of bubbles from HiFi (blue) and ONT (orange) pangenomes are shown on the right of each autosome. There are some fluctuations between the two bubble counts, particularly at chromosome ends, but there are 3.96 and 3.98 bubbles per 100 Kb window for HiFi and ONT respectively. The root mean square difference between them is 0.93.

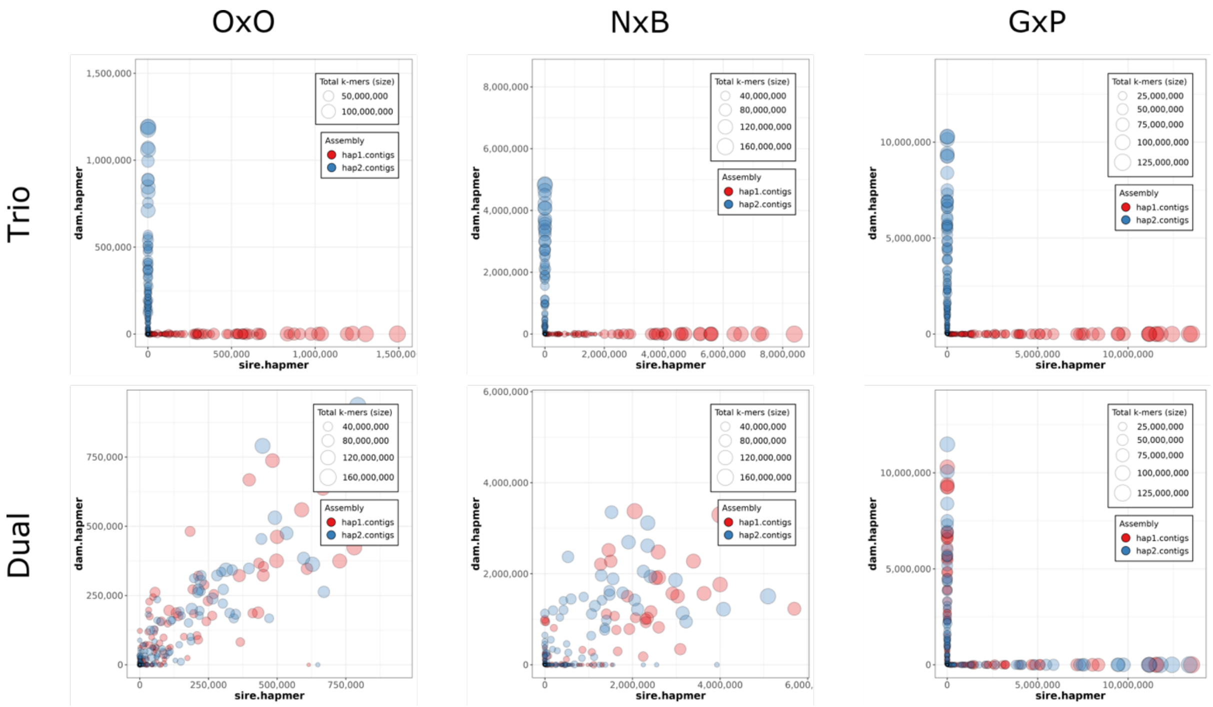

Supplementary Figure 7. Merqury phasing blob plots for OxO, NxB, and GxP hifiasm assemblies using trio or dual approaches. Trio approaches use parental short read information while the dual approach uses a local phasing algorithm in hifiasm using only the F1 HiFi reads. Blobs represent individual contigs, and the axes represent how many specific dam/sire haplotype k-mers (hapmers) the contig contained. Hap1 and hap2 refers to the paternal and maternal haplotypes respectively. Phasing is highly accurate when using parental information (top). Without parental information, increasing heterozygosity allows improved phasing. The mean shortest Euclidian distance for each blob to the diagonal (greater distance indicates a haplotype is more strongly maternal or paternal) increases for the OxO, NxB, and GxP from 3204.5 to 36322.6 and then 138001.4. Hifiasm cannot infer if a contig belongs to the maternal or paternal assembly since there is no parental information but, given enough heterozygosity (e.g., the GxP), is able to consistently phase haplotypes to the contig level.

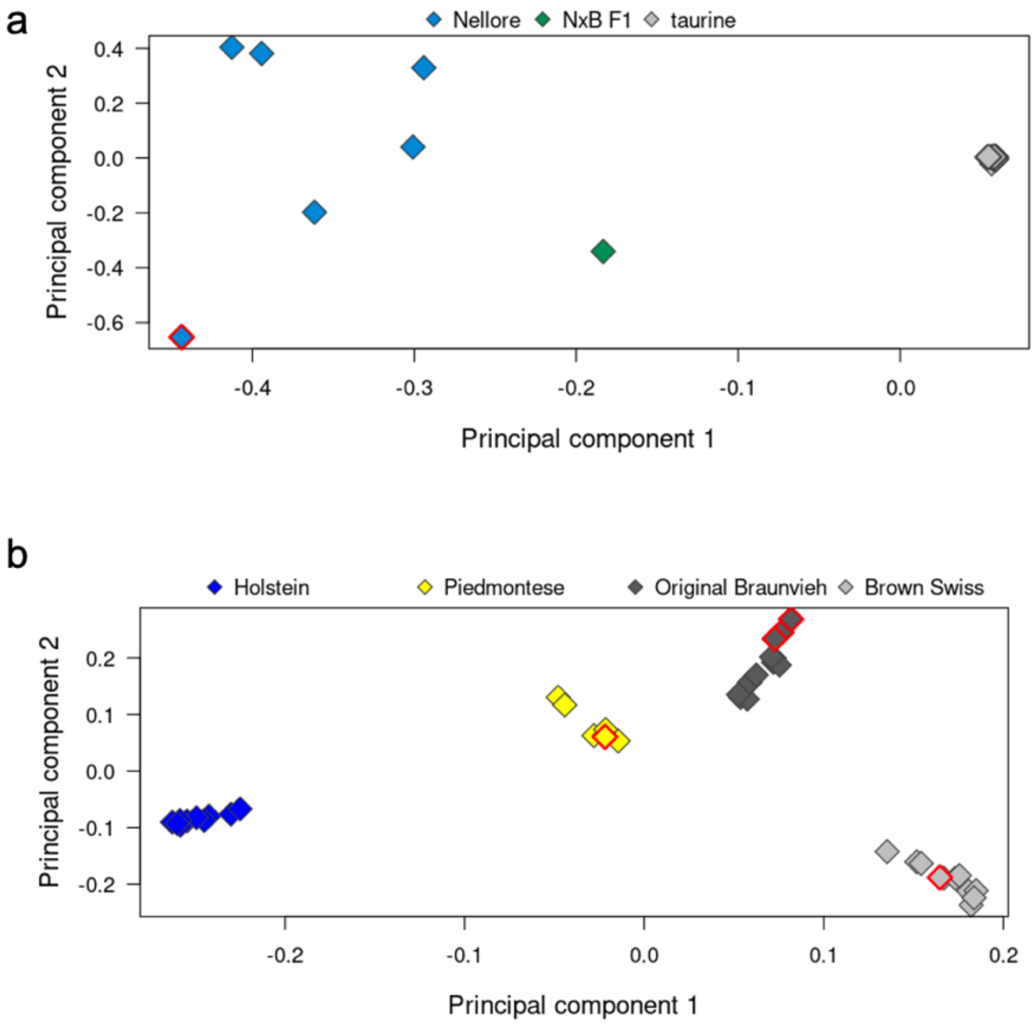

Supplementary Figure 8. PCA of taurine (Holstein, Piedmontese, Brown Swiss, Original Braunvieh), indicine (Nellore) and indicine x taurine (NxB) crosses. Different colors separate individuals by breed. Red framed symbols highlight the purebred animals from the three trios. The PCA was performed for all samples (a) and for the taurine-only (N=41) samples (b). Multi-sample variant calling was done on publicly available whole-genome sequencing data of 10 Brown Swiss (SAMEA5415485, SAMEA7573645, SAMEA7573582, SAMEA7573592,SAMEA7573599, SAMEA5714971, SAMEA6163191, SAMEA7573585, SAMEA6163179, SAMEA6163195), 10 Original Braunvieh (SAMEA4827668, SAMEA5059753, SAMEA5159850, SAMEA6272091, SAMEA4827645, SAMEA4827672, SAMEA6272103, SAMEA6272093, SAMEA5564728, SAMEA5059754), 10 Holstein (SAMEA6528904, SAMEA6528905, SAMEA6528906, SAMEA6528907, SAMEA6528908, SAMEA6528909, SAMEA7015110, SAMEA7015112, SAMEA7015113, SAMEA7015115), 6 Piedmontese (SAMEA5159836, SAMEA19324918, SAMEA33001918, SAMN02941216, SAMN02941219, SAMN02941218) and 5 Nellore (SAMN10486398, SAMN10486400, SAMN10486401, SAMN10486399, SAMN03387027) samples together with short sequencing reads from the OxO and NxB trio as well as the dam of the GxP trio. There were 35.4 million and 20.1 million polymorphic sites along the autosomes used to construct a genomic relationship matrix for the taurine/indicine and taurine-only breeds, respectively.

Supplementary Note 1: Verification of structural variants discovered from the pangenome

After identifying pangenome bubbles overlapping annotated coding sequences, we verified multiple examples by inspecting alignments of long and short sequencing reads against the ARS-UCD1.2 reference sequence. Trio binning and alignment against ARS-UCD1.2 are as described in the Methods. To verify bubbles from the pangenome, we inspected the read alignments at the corresponding positions with the Integrative Genomics Viewer (IGV, [2]).

Recovery of a tandem duplication upstream the group-specific component (*GC*) gene

Alignments of long reads confirmed the presence of a large tandem duplication in the promoter region of GC (ENSBTAG00000013718) in the Brown Swiss and Original Braunvieh haplotype (Supplementary Figure 9). A sequence of 11,780 bp (between 86,949,653 bp and 86,961,433 bp) is duplicated and occurs as between 3 and 4 copies in these haplotypes. This duplication is not present in the gaur, Nellore and Piedmontese haplotype. However, the pangenome revealed two insertions of approximately 178 bp and 730 bp in both the Nellore and gaur haplotypes, that coincide with the tandem duplication (Supplementary Figure 10).

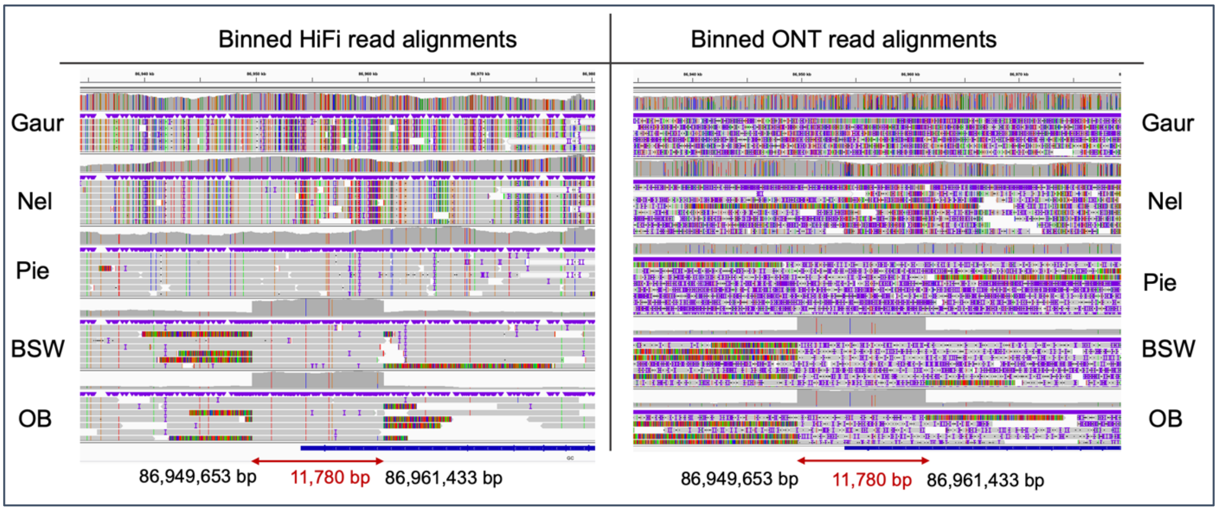

Supplementary Figure 9. Binned HiFi (left) and ONT (right) read alignments against ARS-UCD1.2. The tandem duplication is visible as elevated coverage in BSW and OB between 86,949,653 bp and 86,961,433 bp.

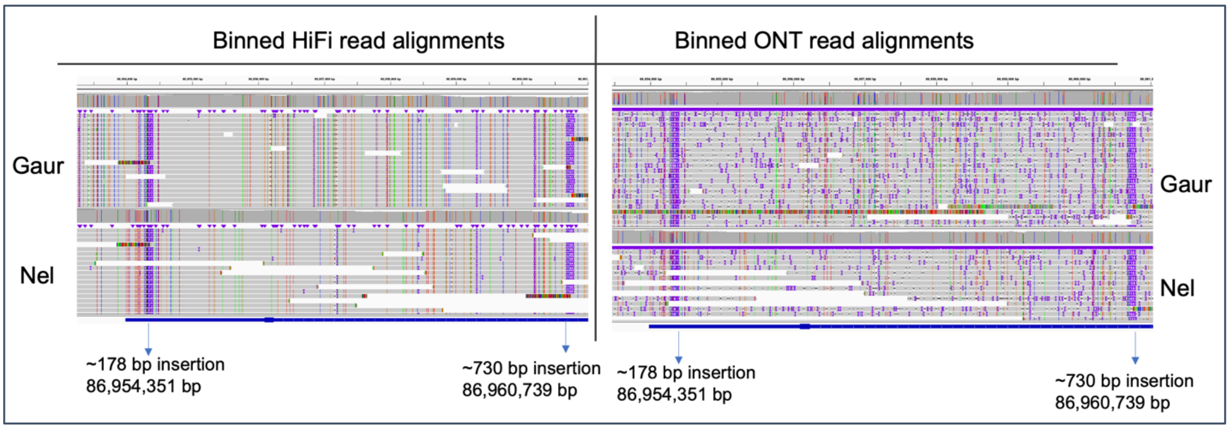

Supplementary Figure 10. Binned HiFi (left) and ONT (right) read alignments against ARS-UCD1.2 indicate two insertions of 178 bp and 730 bp in the gaur and Nellore assemblies.

Structural variation in the promoter region of *ASIP* encoding agouti signaling protein

The coat of the NxB was light at birth but turned almost black with age (Supplementary Figure 11). This darkening with age is frequently observed in Brown Swiss, Original Braunvieh, and Nellore cattle. However, the genetic determinants are largely unknown. A recent study discovered a structural rearrangement in the promoter of *ASIP* (ENSBTAG00000034077) to be associated with variation in coat color in Nellore cattle [3]. The analysis of short read alignments indicated that the Nellore sire indeed is a heterozygous carrier of the 1155 bp deletion and 150 bp insertion of Bov-tA SINE at CHR13:63,599,803–63,600,957 that is associated with darkness of coat [3]. This structural rearrangement is not present in the NxB suggesting it inherited the haplotype without the deletion/insertion from its sire.

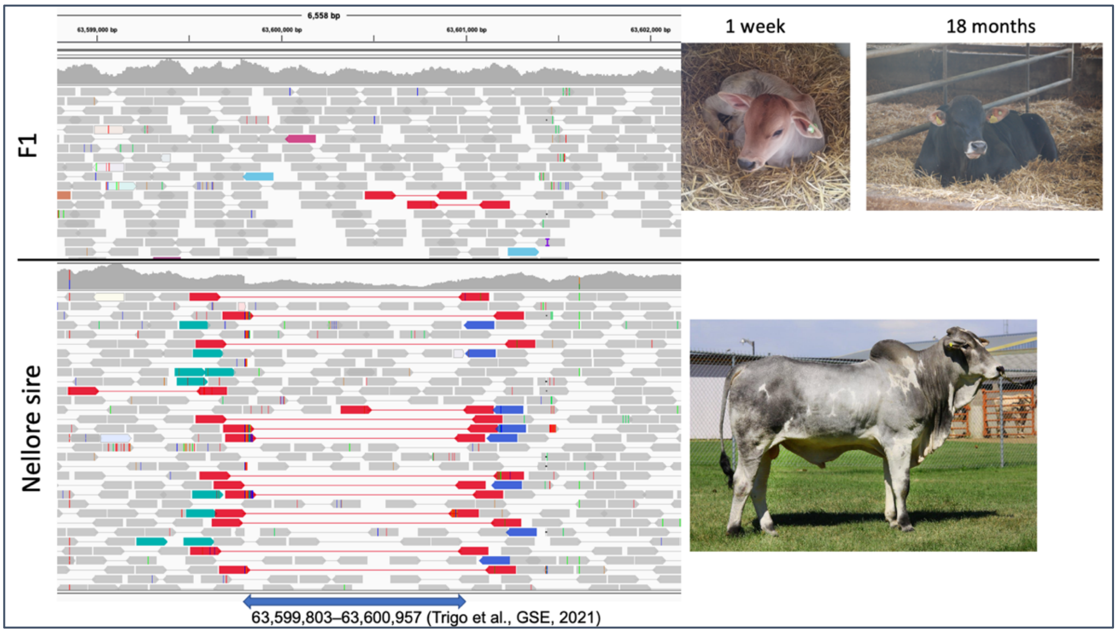

Supplementary Figure 11. IGV screenshots and photographs from the NxB (upper panel) and its Nellore sire (lower panel). Different colors in the IGV screenshot of the Nellore sire represent reads with abnormal insert size indicating the 1155 bp deletion and 150 bp insertion.

We observed extended segments of elevated and reduced coverage in the promoter region of *ASIP* in the haplotype-resolved read alignments (Supplementary Figure 12). Deletion of 8,403 bp sequence between 63,639,803 bp and 63,648,206 bp was visible in all assemblies. In Brown Swiss,

this deletion partly overlapped with a 3,288 bp region of elevated coverage between 63,644,918 bp and 63,648,206 bp. Read coverage varied greatly across the assemblies at a 1278 bp region between 63,629,629 bp and 63,630,907 bp. However, an immediate association between any of the detected large structural variants and coat color was not readily apparent.

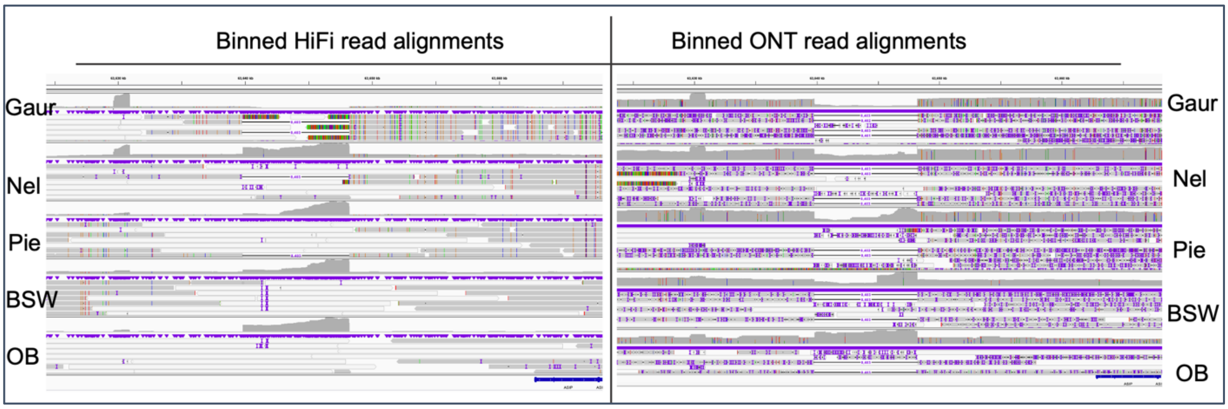

Supplementary Figure 12. Binned HiFi (left) and ONT (right) read alignments against ARS-UCD1.2 indicate various structural rearrangements in the promoter of ASIP.

Expansion of the coding sequence of *QRICH2* relative to ARS-UCD1.2

A pangenome bubble on chromosome 19 overlapped with the fifth exon of *QRICH2* (ENSBTAG00000030173) encoding glutamine rich 2. Inspection of haplotype-resolved alignments revealed various insertions of 30 bp, 90 bp and 120 bp sequence at three distinct locations: 55,423,428 bp, 55,423,639 bp and 55,423,643 bp (Supplementary Figure 13). At 55,423,428, an insertion of a nucleotide sequence «CCAGGCTGGTGTGGTCCAGCCCGGTGCAGC» is visible once in the Pied, BSW and OB assemblies, three times in Nellore, and four times in gaur. Additionally, the same sequence is inserted three times at 55,423,639 bp in the Nellore assembly. In the gaur sequence, a sequence of «CTGGTGCGGTGCAGCCCGGTGCAGGCCAGC» is inserted once at 55,423,642. This expansion of the coding sequence by multiples of 30 bp results in the addition of 10, 50, and 60 amino acids to the glutenine high molecular subunit of bovine QRICH2.

An amino acid motif «QxGxxQPxxx» occurs 16 times between amino acids 713 and 872 in the annotated coding sequence of bovine QRICH2 (ENSBTAT00000065208.2). This motif occurs 17, 21 and 22 times in the haplotype resolved taurine, Nellore, and gaur assemblies. A multi-species alignment [4] of different bovine QRICH2 versions with human orthologs confirms that this amino acid motif is also found in human QRICH2 (Supplementary Figure 14).

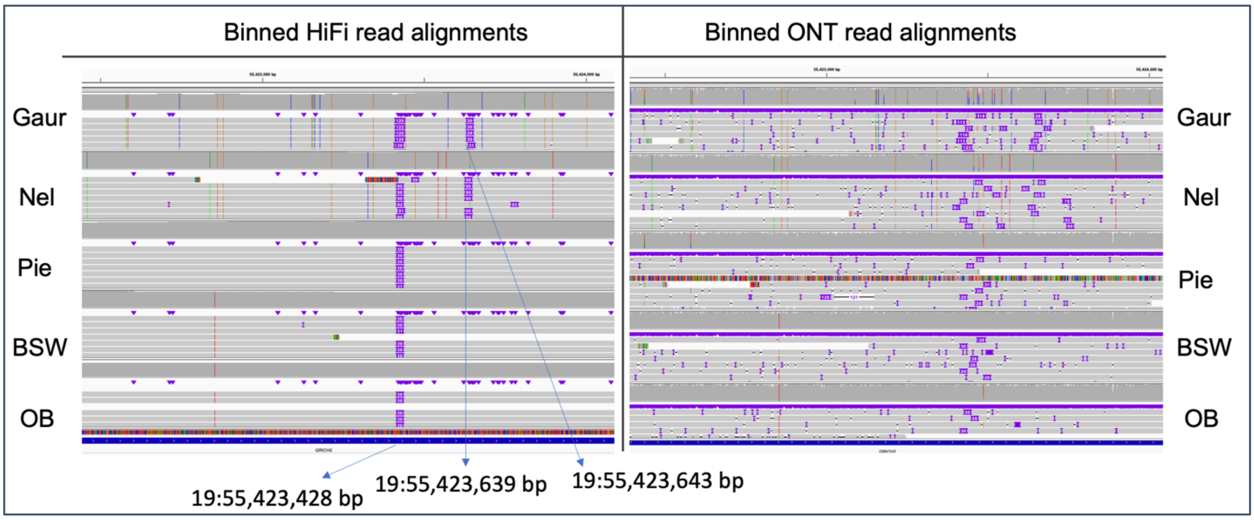

Supplementary Figure 13. Binned HiFi (left) and ONT (right) read alignments against ARS-UCD1.2 indicate multiple instances of duplicated 30 bp sequence in the coding sequence of QRICH2.

Due to the highly repetitive character of the surrounding sequence, the insertion is difficult to discover from short read alignments, as it merely results in increased heterozygosity and allelic imbalance at heterozygous sites (Supplementary Figure 15). The three insertion locations (55,423,428 bp, 55,423,639 bp and 55,423,643 bp) are readily identified by DeepVariant, but only as SNPs, with the first two having lower support than the reference base. Small or structural variant callers, like manta [5] and delly [6], were also unable to identify any of the 30 bp insertions in any of the short read datasets. As such, this variation identified through de novo assemblies and pangenome integration appears completely opaque to short read analysis.

```
QRICH2-206_ENST00000680821.2_Hsa          VDQPGMVQPGRFQRALVQPGAYQPGLVQPGADQIDVVQPGADQHGLVQSGADQSDLAQPG      724
QRICH2-201_ENST00000262765.10_Hsa         VDQPGMVQPGRFQRALVQPGAYQPGLVQPGADQIDVVQPGADQHGLVQSGADQSDLAQPG      558
QRICH2-205_ENST00000636395.1_Hsa          VDQPGMVQPGRFQRALVQPGAYQPGLVQPGADQIDVVQPGADQHGLVQSGADQSDLAQPG      724
QRICH2-201_ENSBTAT00000065208.2_NEL       IGHPGFMQPRMYPRGLVPPRMYHQGLVQPGAGQPGMVQPDIGHPGLVQSGAAQAGAVQPG      720
ENSBIXP00005028105_hybrid                 IGHPGFMQPRMYPRGLVPPRMYHRGLVQPGAGQPGMVQPDIGHPGLVQSGAAQAGAVQPG      730
QRICH2-201_ENSBTAT00000065208.2_Gaur      IGHPGFMQPRMYPRGLVPPRMYHQGLVQPGAGQPGMVQPDIGHPGLVQSGAAQAGVVQPG      720
QRICH2-201_ENSBTAT00000065208.2_ARS       IGHPGFMQPRMYPRGLVPPRMYHQGLVQPGAGQPGMVQPDIGHPGLVQSGAAQAGA----      716
QRICH2-201_ENSBTAT00000065208.2_taurine   IGHPGFMQPRMYPRGLVPPRMYHQGLVQPGAGQPGMVQPDIGHPGLVQSGAAQAGA----      716
                                          :.:**::**  : *.** *  *: *******.* .:***. .: ******* *:.

QRICH2-206_ENST00000680821.2_Hsa          AVQHGLVQPGVDQRGLAQPRADH---------QRGLVPPGADQRGLVQPGADQHGLVQPG      775
QRICH2-201_ENST00000262765.10_Hsa         AVQHGLVQPGVDQRGLAQPRADH---------QRGLVPPGADQRGLVQPGADQHGLVQPG      609
QRICH2-205_ENST00000636395.1_Hsa          AVQHGLVQPGVDQRGLAQPRADH---------QRGLVPPGADQRGLVQPGADQHGLVQPG      775
QRICH2-201_ENSBTAT00000065208.2_NEL       AAQAGAVQPGAGQAGAVQPGAGQAGAVQPGAGQAGAVQPGAVQAGAVQPGVGQPGAVQPG      780
ENSBIXP00005028105_hybrid                 AAQAGAVQPGAGQAGAVQPGA----------GQAGAVQPGAGQAGAVQPGAGQAGAVQPG      780
QRICH2-201_ENSBTAT00000065208.2_Gaur      AAQAGVVQPGAGQAGVIQPGAAQAGAVQPGAGQAGAVQPGAGQAGAVQPGAVQAGAVQPG      780
QRICH2-201_ENSBTAT00000065208.2_ARS       -------------------------VQPGA----------GQAGAVQPGAVQAGAVQPG      740
QRICH2-201_ENSBTAT00000065208.2_taurine   -------------------------VQPGAGQAGAVQPGAGQAGAVQPGAVQAGAVQPG      750
                                                                              * * ****. * * ****

QRICH2-206_ENST00000680821.2_Hsa          VDQHGLAQPGEVQRSLVQPGIVQRGLVQPGAVQRGLVQPGAVQRGLVQPGVDQRGLVQPG      835
QRICH2-201_ENST00000262765.10_Hsa         VDQHGLAQPGEVQRSLVQPGIVQRGLVQPGAVQRGLVQPGAVQRGLVQPGVDQRGLVQPG      669
QRICH2-205_ENST00000636395.1_Hsa          VDQHGLAQPGEVQRSLVQPGIVQRGLVQPGAVQRGLVQPGAVQRGLVQPGVDQRGLVQPG      835
QRICH2-201_ENSBTAT00000065208.2_NEL       VGQAGAVQPGVGQPGAVQPGAAQAGAVQPGAVQTGAVQPGAAQAGVVQPGVGQPGAVQPG      840
ENSBIXP00005028105_hybrid                 VGQPGAVQPGLGQAGAVQPGAGQPGAVQPGAAQAGAVQPGAVQA----------GAVQPG      830
QRICH2-201_ENSBTAT00000065208.2_Gaur      VGQPGAVQPGVGQAGAVQPGVGQPGAVQPGAAQAGAVQPGAVQA----------GAVQPG      830
QRICH2-201_ENSBTAT00000065208.2_ARS       VGQPGAVQPGVGQAGAVQPGVGQPGAVQPGAAQAGAVQPGAVQA----------GA----      786
QRICH2-201_ENSBTAT00000065208.2_taurine   VGQPGAVQPGVGQAGAVQPGVGQPGAVQPGAAQAGAVQPGAVQA----------GA----      796
                                          *.* * .***  * . ****  * * *****.* * *****.*          *

QRICH2-206_ENST00000680821.2_Hsa          AVQRGLVQPGAVQHGLVQPGADQRGLVQPGVDQRGLVQPGVDQRGLVQPGMDQRGLIQPG      895
QRICH2-201_ENST00000262765.10_Hsa         AVQRGLVQPGAVQHGLVQPGADQRGLVQPGVDQRGLVQPGVDQRGLVQPGMDQRGLIQPG      729
QRICH2-205_ENST00000636395.1_Hsa          AVQRGLVQPGAVQHGLVQPGADQRGLVQPGVDQRGLVQPGVDQRGLVQPGMDQRGLIQPG      895
QRICH2-201_ENSBTAT00000065208.2_NEL       AAQAGAVQPGAAQAGVVQPGVGQPGAVQPGAAQAGAVQPGAAQAGVVQPGVGQAGMVQPG      900
ENSBIXP00005028105_hybrid                 AGQPGAVQPGAAQAGVVQPGVGQPGAVQPGAAQAGAVQPGAAQAGVVQPGVGQAGMVQPG      890
QRICH2-201_ENSBTAT00000065208.2_Gaur      AGQAGAVQPGAAQAGVVQPGVGQPGAVQPGAAQAGAVQPGAAQAGVVQPGVGQAGMVQPG      890
QRICH2-201_ENSBTAT00000065208.2_ARS       ------VQPGAAQAGVVQPGVGQPGAVQPGAAQAGAVQPGAAQAGVVQPGVGQAGMVQPG      840
QRICH2-201_ENSBTAT00000065208.2_taurine   ------VQPGAAQAGVVQPGVGQPGAVQPGAAQAGAVQPGAAQAGVVQPGVGQAGMVQPG      850
                                                *****.* *:****..* * ****. * * ****. * *:****:.* *::***

QRICH2-206_ENST00000680821.2_Hsa          ADQPGLVQPGAGQLGMVQPGIGQQGMVQPQADPHGLVQPGAYPLGLVQPGAYLHDLSQSG      955
QRICH2-201_ENST00000262765.10_Hsa         ADQPGLVQPGAGQLGMVQPGIGQQGMVQPQADPHGLVQPGAYPLGLVQPGAYLHDLSQSG      789
QRICH2-205_ENST00000636395.1_Hsa          ADQPGLVQPGAGQLGMVQPGIGQQGMVQPQADPHGLVQPGAYPLGLVQPGAYLHDLSQSG      955
QRICH2-201_ENSBTAT00000065208.2_NEL       AGQPGLVQPQVDQYGLVQPDRGQHGFFQPNISMPGLVPPGAYPPGLVQTGAYPGGLIQPG      960
ENSBIXP00005028105_hybrid                 AGQPGLVQPQVDQYGLVQPDRGQHGFFQPNISMPGLVPPGAYPPGLVQTGAYPGGLIQPG      950
QRICH2-201_ENSBTAT00000065208.2_Gaur      AGQPGLVQPQVDQYGLVQPDRGQHGFFQPNISMPGLVPPGAYPPGLVQTGAYPGGLIQPG      950
QRICH2-201_ENSBTAT00000065208.2_ARS       AGQPGLVQPQVDQYGLVQPDRGQHGFFQPNISMPGLVPPGAYPPGLVQTGAYPGGLIQPG      900
QRICH2-201_ENSBTAT00000065208.2_taurine   AGQPGLVQPQVDQYGLVQPDRGQHGFFQPNISMPGLVPPGAYPPGLVQTGAYPGGLIQPG      910
                                          *.*******  ..* *:***. **:*:.**: .   *** ***** **** ***   .* * *
```

Supplementary Figure 14. Multi-species alignment of QRICH2. Alignment of the different versions of QRICH2 with its human orthologs. Blue background indicates amino acids 713 – 872 of bovine QRICH2 *(ENSBTAT00000065208.2).*

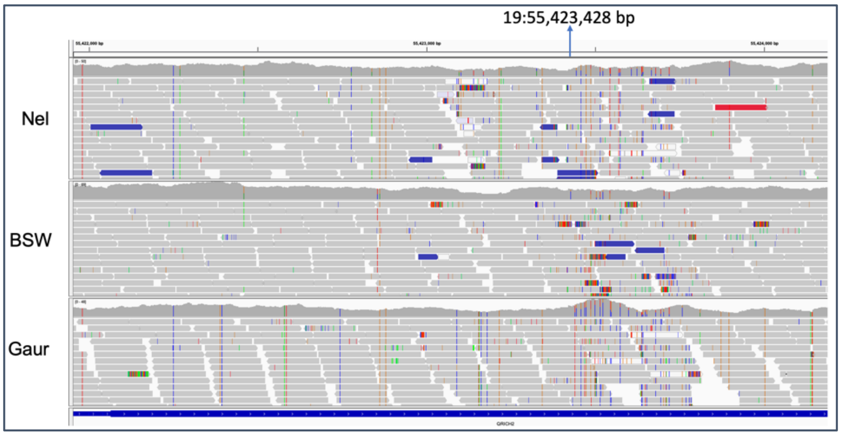

Supplementary Figure 15. Short-read alignments against ARS-UCD1.2 at the fifth exon of QRICH2*. Alignments of short reads from the Nellore sire and BSW dam of NxB, as well as from the gaur sire of GxP against ARS-UCD1.2 at the position where the long read alignments revealed the first insertion of 30 bp sequence.*

Using RNA sequencing data from testis tissue of 76 mature taurine bulls, we confirm that *QRICH2* is transcribed in high abundance (31.52 ± 7.25 transcripts per million) in bovine testes. The fifth exon affected by the expansion of the coding sequence is also regularly expressed (Supplementary Figure 16). However, a drop in sequencing coverage and many soft-clipped reads are visible at the region that harbors the repetitive motif likely indicating that increased ambiguity and incomplete reference

sequence impairs read mapping (Supplementary Figure 17). The raw RNA sequencing data used to investigate transcript abundance is available via [7] from the European Nucleotide Archive at primary accession via PRJEB46995.

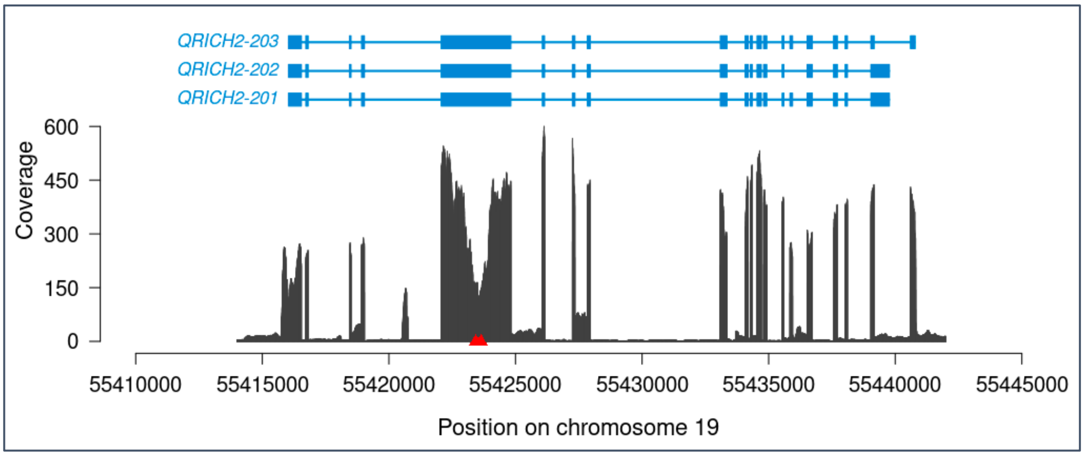

Supplementary Figure 16. Average coverage of RNA sequencing reads in testes of 76 mature bulls. The red diamonds represent the locations (55,423,428 bp, 55,423,639 bp, 55,423,643 bp) of the inserted sequences affecting the coding sequence of the fifth exon. Blue color represents the exon-intron structure of three QRICH2 isoforms.

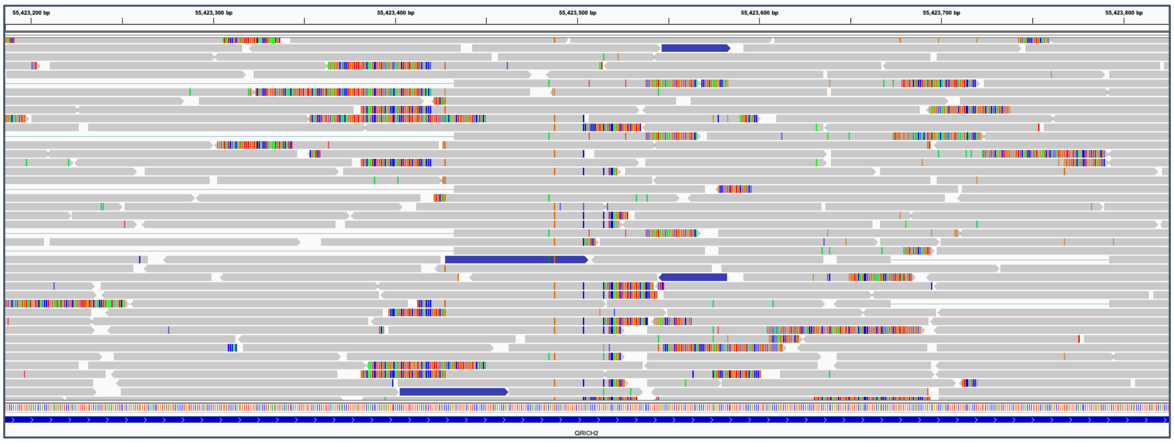

Supplementary Figure 17. Representative IGV screenshot of RNA sequencing data from testis tissue of a Brown Swiss bull aligned against ARS-UCD1.2 reference sequence at a region (Chr19:55,423,200 – 55,423,800) within the fifth exon of QRICH2. Many reads with soft clipped bases are indicative for the expansion of the coding sequence compared to ARS-UCD1.2. The raw RNA sequencing data underpinning this alignment are available at accession SAMEA9540538.

Long read alignments of other publicly available long read bovid samples reveal additional expansions relative to ARS-UCD1.2 for yak, water buffalo, bison, and Highland cattle, but not in Simmental cattle (Supplementary Figure 18). The presence of multiple insertions relative to ARS-UCD1.2 and other taurine assemblies in different bovid species suggests loss of *QRICH2* coding sequence in the taurine lineage. However, the error-prone CLR and ONT alignments are unable to cleanly resolve this challenging region. Alignments of long reads from Dominette, the cow on which ARS-UCD1.2 is based, indicate she may have carried the putative 30 bp deletion in the heterozygous state, or could have been an assembler/polishing collapse due to the high sequence similarity of these repeats. High-quality assemblies and long read alignments from additional taurine breeds and distantly related bovid samples are required to clearly disentangle the evolutionary relationship surrounding the *QRICH2* SVs.

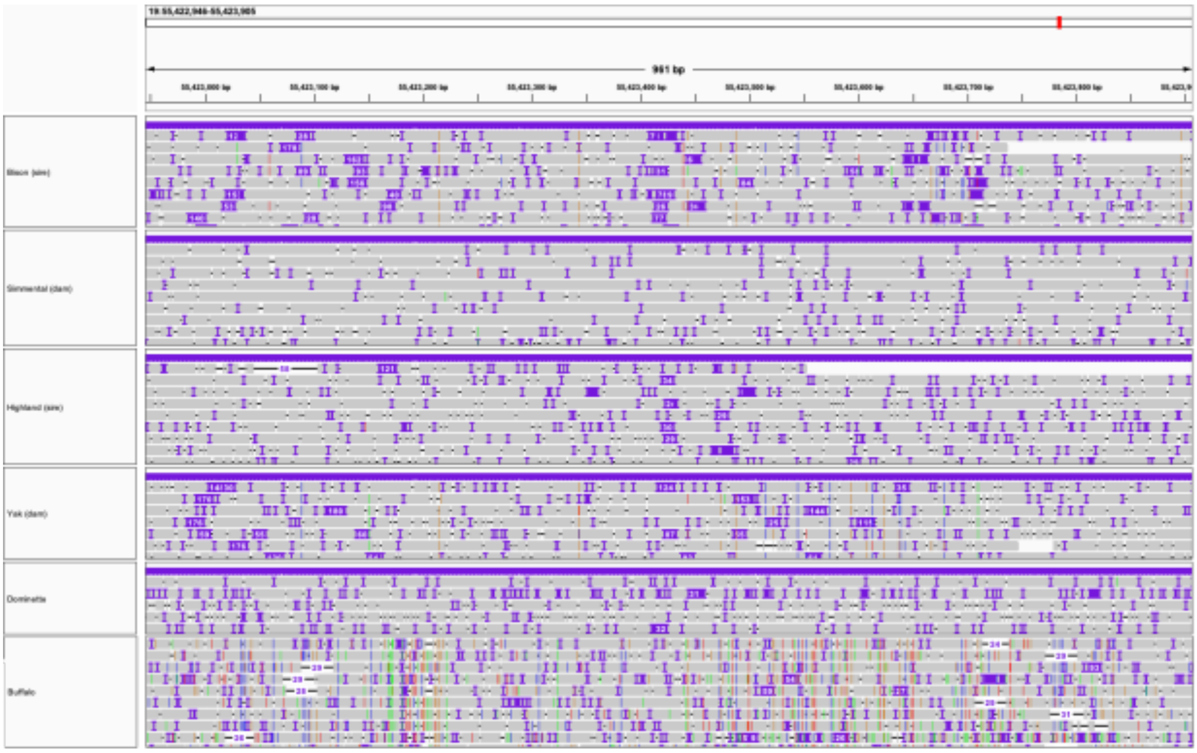

Supplementary Figure 18. SVs in QRICH2 *across other bovids. Alignments of trio binned ONT reads from a bison x Simmental cross* [8], [9]*, trio binned CLR reads from a Highland x yak* [10] *cross, and CLR reads from a water buffalo* [11] *reveal similar patterns of insertions relative to ARS-UCD1.2. Alignments of CLR reads from Dominette* [12] *also indicate a potential insertion at the expected coordinate.*

Deletion of *TAS2R46* and *ENSBTAG00000001761*

A pangenome bubble on chromosome 5 overlapped with *TAS2R46* (ENSBTAG00000030471) and *ENSBTAG00000001761* encoding taste 2 receptor member 46 and olfactory receptor family 6 subfamily AA member 1, respectively. Inspection of long read alignments confirmed deletion of 17,017 bp in the gaur assembly between 98,587,384 and 98,604,401 bp encompassing the entire coding sequences of *TAS2R46* and ENSBTAG00000001761 (Supplementary Figure 19). This deletion was not detected in the other haplotype-resolved assemblies.

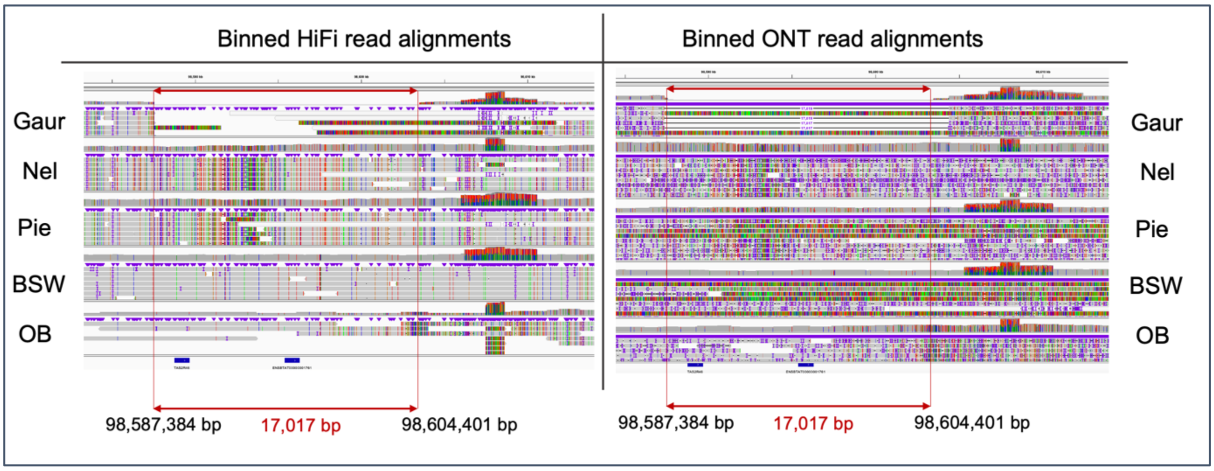

Supplementary Figure 19. Binned HiFi (left) and ONT (right) read alignments against ARS-UCD1.2 indicate deletion of 17 kb in the gaur assembly. Red color highlights the coordinates of the deletion in gaur encompassing two genes (blue tracks at the bottom).

Coverage of short read alignments from the gaur sire verifies the presence of the deletion (Supplementary Figure 20). The gaur sire carries the deletion in the heterozygous state suggesting within-species variability and the transmission of the haplotype carrying the deletion to the F1.

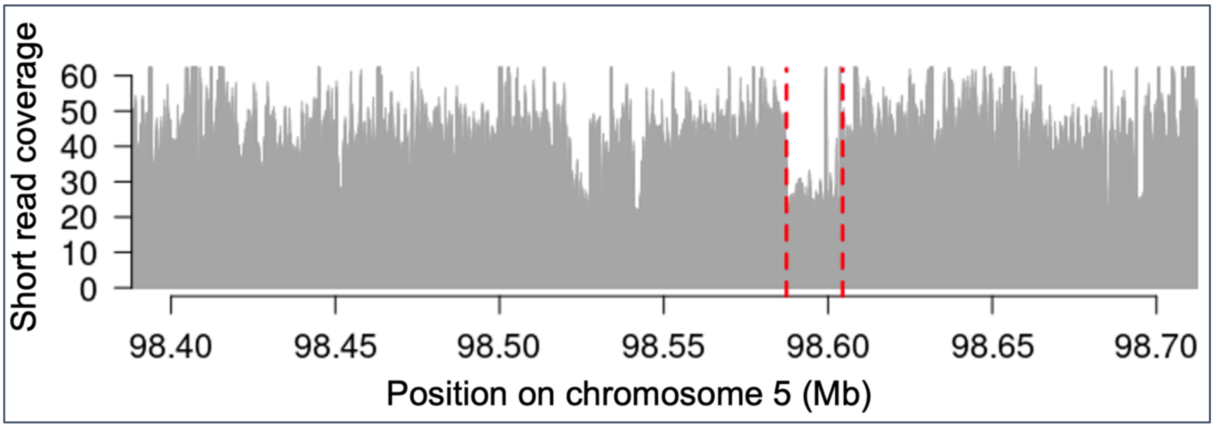

Supplementary Figure 20. Short read alignments from the gaur sire against ARS-UCD1.2 confirm the deletion of a 17 kb segment on chromosome 5. Red dotted lines indicate the location of deletion. Short read coverage at the deletion is approximately half the average coverage, indicating that the gaur is heterozygous carrier of the deletion.

Duplication of *HSPA1A*

A pangenome bubble at chromosome 23 overlapped *HSPA1A* (ENSBTAG00000025441) encoding heat shock protein family A (Hsp70) member 1A. We observed elevated coverage at a 2,294 bp segment between 27,520,653 bp and 27,522,947 bp and multiple soft-clipped reads extending the seemingly duplicated region in all haplotype-resolved alignments (Supplementary Figure 21). The region of elevated coverage encompasses the 5'UTR and the entire exon of *HSPA1A*. Manual inspection and assembly of the soft clipped bases enabled reconstructing an insertion of approximately 11 kb insertion (Supplementary Figure 22). The 11 Kb insertion can also be identified through calling structural variants with the long reads, as confirmed by pbsv (https://github.com/pacificbiosciences/pbsv). Elevated coverage at the 2,294 bp segment encompassing *HSPA1A* is likely due to reads from *HSPA1B* that cannot align properly, as *HSPA1B* is missing in the current *Bos taurus* reference genome [13]. The pangenome region and respective repeat analysis is shown in Supplementary Figure 23.

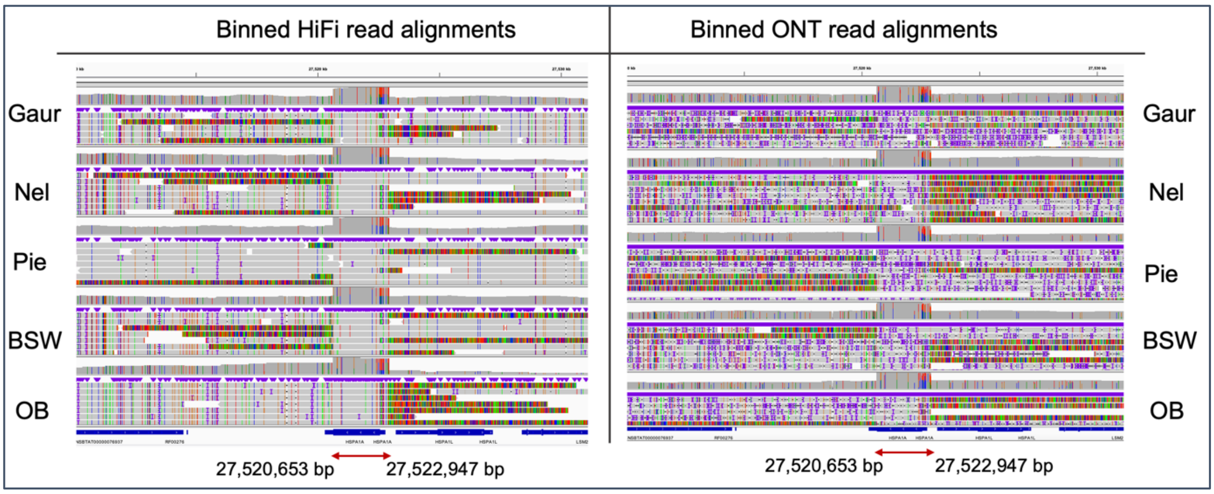

Supplementary Figure 21. Binned HiFi (left) and ONT (right) read alignments against ARS-UCD1.2 as well as the corresponding coverage tracks indicate duplication of a 2.3 kb segment encompassing HSPA1A *in all assemblies.*

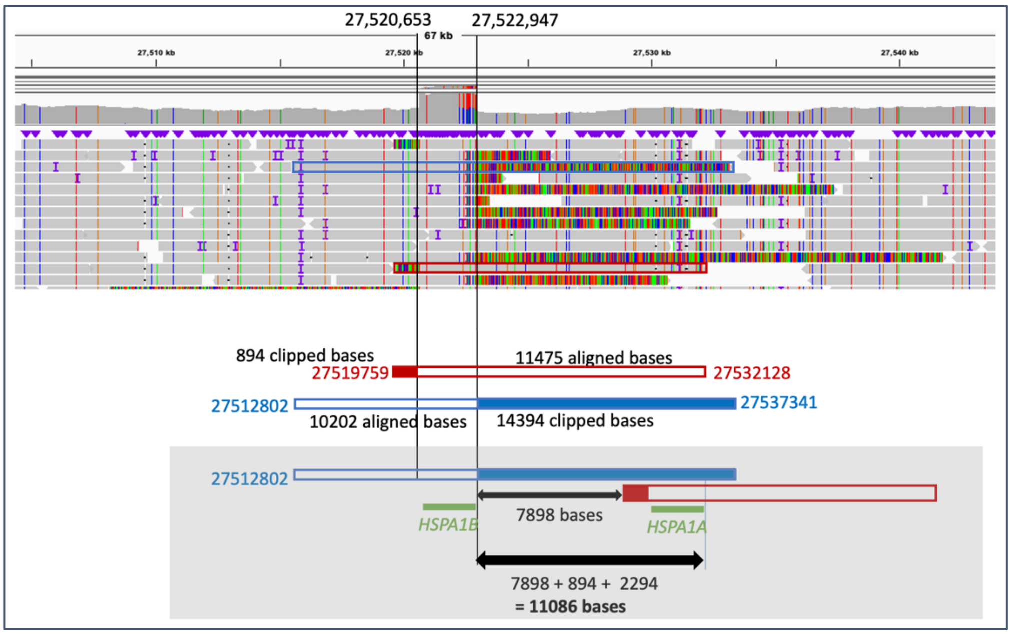

Supplementary Figure 22. Recovery of an approximately 11 kb insertion in the Nellore haplotype from manual assembly of reads with soft-clipped bases.

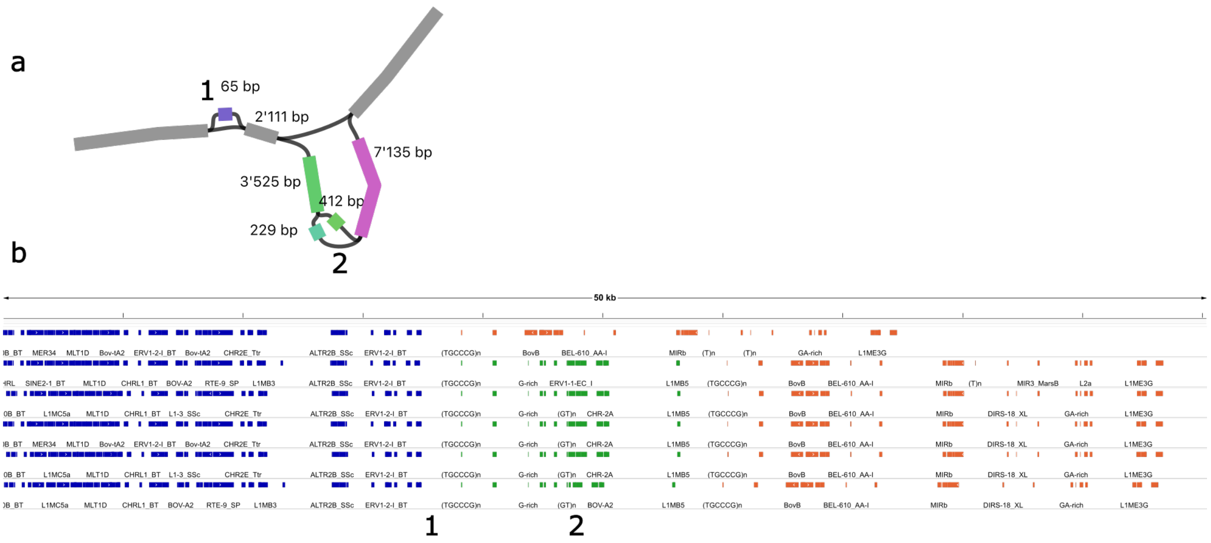

Supplementary Figure 23. The hifiasm pangenome region around HSPA1A *(located within the orange repeats) predicts an 11 Kb insertion, which contains* HSPA1B *(located within the green repeats). The Original Braunvieh assembly contains a 65 bp insertion (marked as "1"), while the gaur assembly contains a 200 bp deletion (marked as "2"). The hifiasm gaur haplotype path through the insertion is approximately 10,889 bp, while the other haplotype path is 11,072 bp, in strong agreement with the soft-clipped read estimation from Supplementary Figure 22.*

Insertion and deletion of 84 bp in the coding sequence of *PRDM9*

The pangenome graph contained a bubble on chromosome 1 with an 84 bp deletion that overlapped the eleventh exon of *PRDM9* (ENSBTAG00000004538) encoding PR domain containing 9. Read alignments confirm the deletion of 84 bp («GCCCACACTCCCCGC AAACATAGGGCTTCTCCCCTGTGTGTGTCCTCTGGTGTGTGATGAGAGTGGACTTCTGATTGAAGCTTT») between 157,543,126 bp and 157,546,209 bp in the gaur and Nellore assemblies (Supplementary Figure 24). In the paternal Original Braunvieh haplotype, an insertion of 84 bp («CCCACACTCCCCGCAAACATAGGGCTTCTCCCCTGTGTGTGTCCTCTGGTGTGTGATGAGACGGCCCTTCTGACTGAAGCTTTG») occurred at 157,546,378 bp.

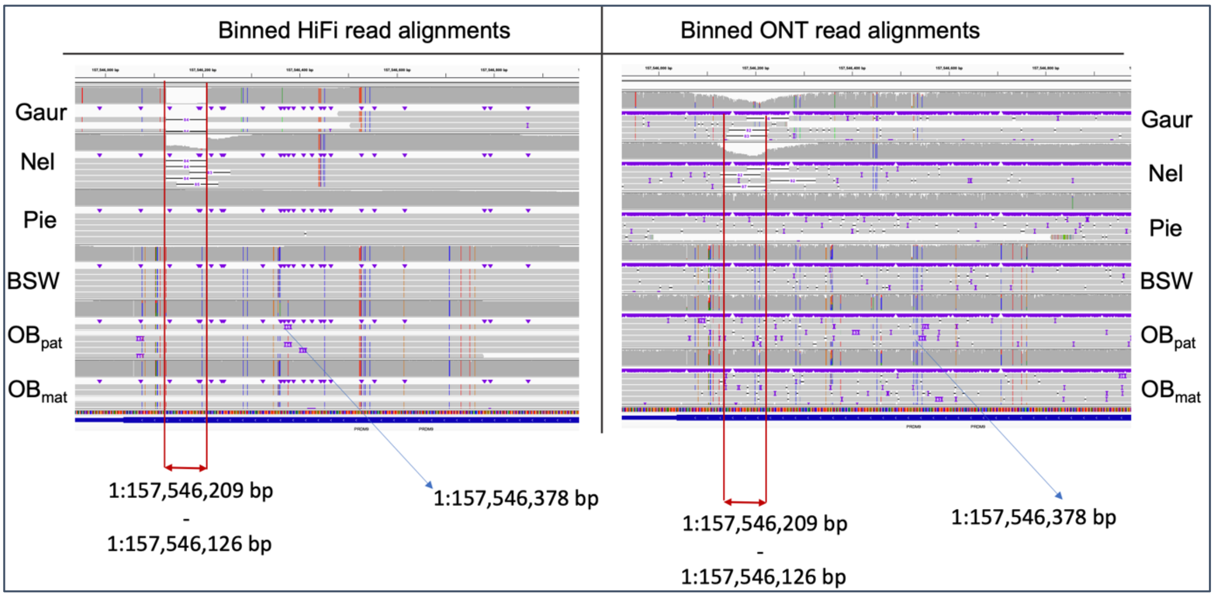

Supplementary Figure 24. Binned HiFi (left) and ONT (right) read alignments against ARS-UCD1.2 as well as the corresponding coverage tracks indicate deletion and insertions of 84 bp in the eleventh exon of PRDM9.

Due to the repetitive nature of the sequence encoding the zinc fingers, this variation is difficult to resolve using short read alignments (Supplementary Figure 25).

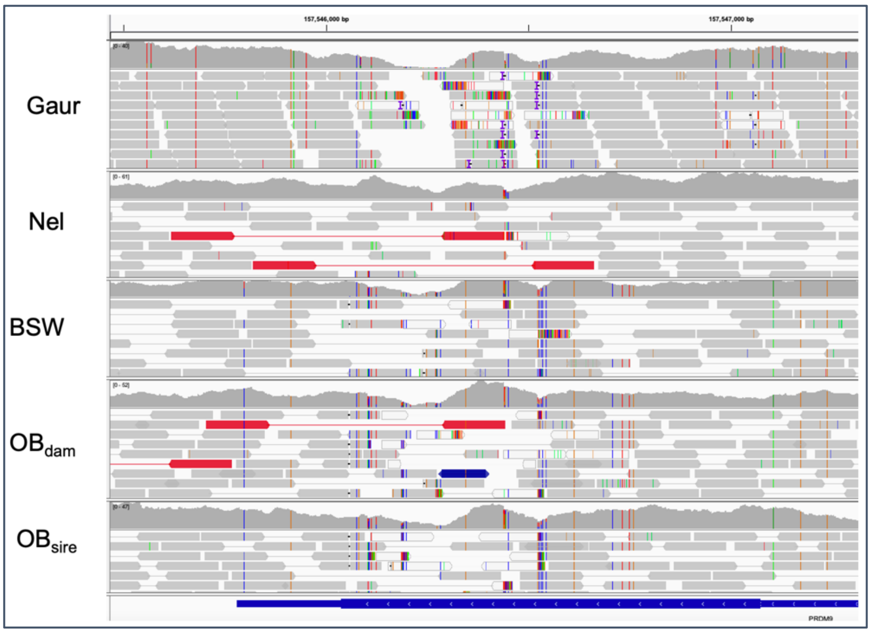

Supplementary Figure 25. Alignments of parental short reads against ARS-UCD1.2 at the eleventh exon of PRDM9. Red colored symbols and low coverage indicate the deletion in the gaur and Nellore sires.

Supplementary References

[1] A. Leonard, “Bovine pangenome assemblies,” Jan. 2022, doi: 10.5281/ZENODO.5906579.

[2] J. T. Robinson *et al.*, “Integrative Genomics Viewer,” *Nat. Biotechnol.*, vol. 29, no. 1, p. 24, Jan. 2011, doi: 10.1038/NBT.1754.

[3] B. B. Trigo *et al.*, “Variants at the ASIP locus contribute to coat color darkening in Nellore cattle,” *Genet. Sel. Evol. 2021 531*, vol. 53, no. 1, pp. 1–12, Apr. 2021, doi: 10.1186/S12711-021-00633-2.

[4] F. Sievers and D. G. Higgins, “Clustal Omega for making accurate alignments of many protein sequences,” *Protein Sci.*, vol. 27, no. 1, pp. 135–145, Jan. 2018, doi: 10.1002/pro.3290.

[5] X. Chen *et al.*, “Manta: rapid detection of structural variants and indels for germline and cancer sequencing applications,” *Bioinformatics*, vol. 32, no. 8, pp. 1220–1222, Apr. 2016, doi: 10.1093/BIOINFORMATICS/BTV710.

[6] T. Rausch, T. Zichner, A. Schlattl, A. M. Stütz, V. Benes, and J. O. Korbel, “DELLY: structural variant discovery by integrated paired-end and split-read analysis,” *Bioinformatics*, vol. 28, no. 18, pp. i333–i339, Sep. 2012, doi: 10.1093/BIOINFORMATICS/BTS378.

[7] N. K. Kadri, X. M. Mapel, and H. Pausch, “The intronic branch point sequence is under strong evolutionary constraint in the bovine and human genome,” *Commun. Biol. 2021 41*, vol. 4, no. 1, pp. 1–13, Oct. 2021, doi: 10.1038/s42003-021-02725-7.

[8] M. P. Heaton *et al.*, “A Reference Genome Assembly of Simmental Cattle, Bos taurus taurus,” *J. Hered.*, vol. 112, no. 2, pp. 184–191, Mar. 2021, doi: 10.1093/JHERED/ESAB002.

[9] J. Oppenheimer *et al.*, “A Reference Genome Assembly of American Bison, Bison bison bison,” *J. Hered.*, vol. 112, no. 2, pp. 174–183, Mar. 2021, doi: 10.1093/JHERED/ESAB003.

[10] E. S. Rice *et al.*, “Continuous chromosome-scale haplotypes assembled from a single interspecies F1 hybrid of yak and cattle,” *Gigascience*, vol. 9, no. 4, pp. 1–9, Apr. 2020, doi: 10.1093/GIGASCIENCE/GIAA029.

[11] W. Y. Low *et al.*, “Chromosome-level assembly of the water buffalo genome surpasses human and goat genomes in sequence contiguity,” *Nat. Commun. 2019 101*, vol. 10, no. 1, pp. 1–11, Jan. 2019, doi: 10.1038/s41467-018-08260-0.

[12] B. D. Rosen *et al.*, “De novo assembly of the cattle reference genome with single-molecule sequencing,” *Gigascience*, vol. 9, no. 3, pp. 1–9, Mar. 2020, doi: 10.1093/gigascience/giaa021.

[13] M. F. Suqueli García, M. A. Castellote, S. E. Feingold, and P. M. Corva, “Characterization of a deletion in the Hsp70 cluster in the bovine reference genome,” *Anim. Genet.*, vol. 48, no. 4, pp. 377–385, Aug. 2017, doi: 10.1111/age.12561.
