# Supplementary material for: Structural variant-based pangenome construction has low sensitivity to variability of haplotype-resolved bovine assemblies
Source: Nat Commun. 2022 May 31;13:3012. doi: 10.1038/s41467-022-30680-2 (PMC9156671; doi:10.1038/s41467-022-30680-2)
Supplement: Supplementary file 3 — Description of Additional Supplementary Files [file 41467_2022_30680_MOESM3_ESM.pdf]

Description of Additional Supplementary Files

File Name: Supplementary Data 1
Description: Full set of genes overlapped by hifiasm or Shasta pangenome SVs. ARS-UCD1.2 coordinates for the start and end of the overlapped coding sequence region are given for both hifiasm (H) and Shasta (S) pangenomes, along with the number of coding sequence regions overlapped, the mean length of the pangenome bubbles, and the mean number of non-reference paths through the bubble. OMIA indicates if the gene is present in the OMIA database and pLI is the pLI score of the gene (0 if not available).
