# Supplementary material for: Structural variant-based pangenome construction has low sensitivity to variability of haplotype-resolved bovine assemblies
Source: Nat Commun. 2022 May 31;13:3012. doi: 10.1038/s41467-022-30680-2 (PMC9156671; doi:10.1038/s41467-022-30680-2)
Supplement: Supplementary file 5 — Reporting Summary [file 41467_2022_30680_MOESM5_ESM.pdf]

nature portfolio

Corresponding author(s): Alexander Leonard, Ben Rosen, Hubert Pausch

Last updated by author(s): Apr 28, 2022

Reporting Summary

Nature Portfolio wishes to improve the reproducibility of the work that we publish. This form provides structure for consistency and transparency in reporting. For further information on Nature Portfolio policies, see our Editorial Policies and the Editorial Policy Checklist.

Statistics

For all statistical analyses, confirm that the following items are present in the figure legend, table legend, main text, or Methods section.

n/a	Confirmed	
☐	☒	The exact sample size (*n*) for each experimental group/condition, given as a discrete number and unit of measurement
☐	☒	A statement on whether measurements were taken from distinct samples or whether the same sample was measured repeatedly
☐	☒	The statistical test(s) used AND whether they are one- or two-sided<br>*Only common tests should be described solely by name; describe more complex techniques in the Methods section.*
☒	☐	A description of all covariates tested
☒	☐	A description of any assumptions or corrections, such as tests of normality and adjustment for multiple comparisons
☐	☒	A full description of the statistical parameters including central tendency (e.g. means) or other basic estimates (e.g. regression coefficient) AND variation (e.g. standard deviation) or associated estimates of uncertainty (e.g. confidence intervals)
☐	☒	For null hypothesis testing, the test statistic (e.g. *F*, *t*, *r*) with confidence intervals, effect sizes, degrees of freedom and *P* value noted<br>*Give P values as exact values whenever suitable.*
☒	☐	For Bayesian analysis, information on the choice of priors and Markov chain Monte Carlo settings
☒	☐	For hierarchical and complex designs, identification of the appropriate level for tests and full reporting of outcomes
☒	☐	Estimates of effect sizes (e.g. Cohen's *d*, Pearson's *r*), indicating how they were calculated

Our web collection on statistics for biologists contains articles on many of the points above.

Software and code

Policy information about availability of computer code

Data collection	Standard code from sequencing providers (PacBio, Illumina, ONT) was used to gather sequencing data.
Data analysis	Custom scripts and details on software are freely available at https://github.com/AnimalGenomicsETH/bovine-assembly. Sequence filtering done with fastp (https://github.com/OpenGene/fastp) version 0.21.1. Read trio binning (and assembly) done with Canu (https://github.com/marbl/canu) version e0d6bb0. Assembly also done with hifiasm (https://github.com/chhylp123/hifiasm) version 0.15.3-r339, peregrine (https://github.com/cschin/Peregrine) version main:2aefc14+, Shasta (https://github.com/chanzuckerberg/shasta) version 0.7, Flye (https://github.com/fenderglass/Flye) version 2.8.3-b1725, and Raven (https://github.com/lbcb-sci/raven) version 1.5.0. Polishing was done with PEPPER (https://github.com/kishwarshafin/pepper) versions 0.1 and 0.4.1, minimap2 (https://github.com/lh3/minimap2) version 2.19-r1059-dirty, DeepVariant (https://github.com/google/deepvariant) version 1.1.0, merfin (https://github.com/arangrhie/merfin) version 1331fa5. Scaffolding was done with RagTag (https://github.com/malonge/RagTag) verison 2.0.1. Quality was assessed with BUSCO (https://gitlab.com/ezlab/busco) version 5.1.2 using metaeuk (https://github.com/soedinglab/metaeuk) version 9dee7a7, merqury (https://github.com/marbl/merqury) version 1.3, meryl (https://github.com/marbl/meryl) version r953, and calN50.js (https://github.com/lh3/calN50). Repeats were identified with RepeatMasker (http://www.repeatmasker.org) version 4.1.1. Sequence operations were done with seqtk (https://github.com/lh3/seqtk) version 1.3-r115-dirty. Pangenomes were constructed with minigraph (https://github.com/lh3/minigraph) version 0.15-r426, and visualised with Bandage (https://github.com/rrwick/Bandage) version 0.8.1. Miscellaneous analysis was done with samtools (https://github.com/samtools/samtools) version 1.12, bcftools (https://github.com/samtools/bcftools) version 1.12, IGV (https://software.broadinstitute.org/software/igv/) version 2.11.0, bedtools (https://github.com/arq5x/bedtools2) version 2.30.0, gfatools (https://github.com/lh3/gfatools) version 0.5, bwa-mem2 (https://github.com/bwa-mem2/bwa-mem2) version 2.2.1, Ratatosk (https://github.com/DecodeGenetics/Ratatosk) version 0.1, pyBigWig (https://github.com/deeptools/pyBigWig) version 0.3.18, vcf-kit (https://github.com/AndersenLab/VCF-kit) version 0.2.9, megadepth (https://github.com/ChristopherWilks/megadepth) version 1.1.0c, GLNexus (https://github.com/dnanexus-rnd/GLnexus) version 1.3.1, and UpSetPlot (https://github.com/jnothman/UpSetPlot) version 0.6.0.

For manuscripts utilizing custom algorithms or software that are central to the research but not yet described in published literature, software must be made available to editors and reviewers. We strongly encourage code deposition in a community repository (e.g. GitHub). See the Nature Portfolio guidelines for submitting code & software for further information.

Data

Policy information about availability of data

All manuscripts must include a data availability statement. This statement should provide the following information, where applicable:

- Accession codes, unique identifiers, or web links for publicly available datasets
- A description of any restrictions on data availability
- For clinical datasets or third party data, please ensure that the statement adheres to our policy

HiFi reads for the OxO and NxB F1s are available in the ENA database at the study accession PRJEB42335 under sample accession SAMEA7759028 (https://www.ebi.ac.uk/ena/browser/view/SAMEA7759028) and SAMEA7765441 (https://www.ebi.ac.uk/ena/browser/view/SAMEA7765441).

ONT reads for the OxO and NxB are available in the ENA database at the study accession PRJEB42335 under sample accession SAMEA10017983 (https://www.ebi.ac.uk/ena/browser/view/SAMEA10017983) and SAMEA10017982 (https://www.ebi.ac.uk/ena/browser/view/SAMEA10017982).

Short reads for the OxO and NxB are available in the ENA database under accession number SAMEA9986200 (https://www.ebi.ac.uk/ena/browser/view/SAMEA9986200) and SAMEA7589752 (https://www.ebi.ac.uk/ena/browser/view/SAMEA7589752). Parental short reads are available in the ENA database at SAMEA9986201 (https://www.ebi.ac.uk/ena/browser/view/SAMEA9986201) & SAMEA9986199 (https://www.ebi.ac.uk/ena/browser/view/SAMEA9986199) (OxO) and at SAMEA6163185 (https://www.ebi.ac.uk/ena/browser/view/SAMEA6163185) & SAMEA9533783 (https://www.ebi.ac.uk/ena/browser/view/SAMEA9533783) (NxB).

Long and short read sequencing data for the GxP trio are available in the ENA database at the study accession PRJEB48481 under secondary accessions SAMEA10563833 (https://www.ebi.ac.uk/ena/browser/view/SAMEA10563833), SAMEA10563834 (https://www.ebi.ac.uk/ena/browser/view/SAMEA10563834), and SAMEA10563835 (https://www.ebi.ac.uk/ena/browser/view/SAMEA10563835).

The OMIA database is available online (https://www.omia.org/home/) as well as pLI scores in human orthologues (ftp://ftp.broadinstitute.org/pub/ExAC_release/release1/manuscript_data/forweb_cleaned_exac_r03_march16_z_data_pLI.txt.gz). The generated assemblies are available online (https://doi.org/10.5281/ZENODO.5906579).

Field-specific reporting

Please select the one below that is the best fit for your research. If you are not sure, read the appropriate sections before making your selection.

☒ Life sciences ☐ Behavioural & social sciences ☐ Ecological, evolutionary & environmental sciences

For a reference copy of the document with all sections, see nature.com/documents/nr-reporting-summary-flat.pdf

Life sciences study design

All studies must disclose on these points even when the disclosure is negative.

Sample size	Sequencing was collected to at least 15x coverage for short reads, 30x for HiFi, and 30x for ONT, which was sufficient to perform assembly and associated analysis based on established recommendations in the literature. This was done for the F1 of three bovine trios, corresponding to 6 haplotypes (5 unique breeds/species), which were the three crosses available at the time of this study and are representative of possible heterozygosities in future studies.
Data exclusions	HiFi reads were filtered to remove reads below 1 Kb in length or below quality value of 20. Shasta assemblies were run on subsets of data involving reads longer than 10 Kb or 30 Kb. Otherwise no data was excluded during analysis.
Replication	Sequencing subsets were randomly sampled three times when coverage was below 20x. Pangenomes were constructed 20 times with different random orderings of input assemblies. In all cases, observed variation was minor and did not affect any conclusions.
Randomization	This study did not include experimental groups
Blinding	This study did not involve allocating samples to groups

Reporting for specific materials, systems and methods

We require information from authors about some types of materials, experimental systems and methods used in many studies. Here, indicate whether each material, system or method listed is relevant to your study. If you are not sure if a list item applies to your research, read the appropriate section before selecting a response.

Materials & experimental systems

n/a	Involved in the study
☒	☐ Antibodies
☒	☐ Eukaryotic cell lines
☒	☐ Palaeontology and archaeology
☐	☒ Animals and other organisms
☒	☐ Human research participants
☒	☐ Clinical data
☒	☐ Dual use research of concern

Methods

n/a	Involved in the study
☒	☐ ChIP-seq
☒	☐ Flow cytometry
☒	☐ MRI-based neuroimaging

Animals and other organisms

Policy information about studies involving animals; ARRIVE guidelines recommended for reporting animal research

Laboratory animals	No laboratory animals were involved in this work.
Wild animals	This study did not involve wild animals
Field-collected samples	Cows from the Original Braunvieh (O, Bos taurus taurus), Brown Swiss (B, Bos t. t.), and Piedmontese (P, Bos t. t.) breeds were inseminated with semen samples from Original Braunvieh (Bos t. t.), Nellore (N, Bos t. indicus) , and gaur (G, Bos gaurus) sires, respectively. A female (OxO) and a male (NxB) calf were delivered at term and samples were collected at approximately 4 weeks. A female fetus (GxP) cross was collected by cesarean section at 118 days of gestation.
Ethics oversight	The sampling of blood from the NxB and OxO trios was approved by the veterinary office of the Canton of Zurich (animal experimentation permit ZH 200/19). All GxP protocols were approved by the Institutional Animal Care and Use Committee (IACUC) of the University of Nebraska–Lincoln, an AAALAC International Accredited institution (IACUC Project ID 1697). Gaur semen was collected and preserved by Omaha's Henry Doorly Zoo and all protocols were reviewed and approved by their IACUC in 1992.

Note that full information on the approval of the study protocol must also be provided in the manuscript.
